# Supplementary material for: Mapping and phasing of structural variation in patient genomes using nanopore sequencing
Source: Nat Commun. 2017 Nov 6;8:1326. doi: 10.1038/s41467-017-01343-4 (PMC5673902; doi:10.1038/s41467-017-01343-4)
Supplement: Supplementary file 1 — Supplementary Information [file 41467_2017_1343_MOESM1_ESM.pdf]

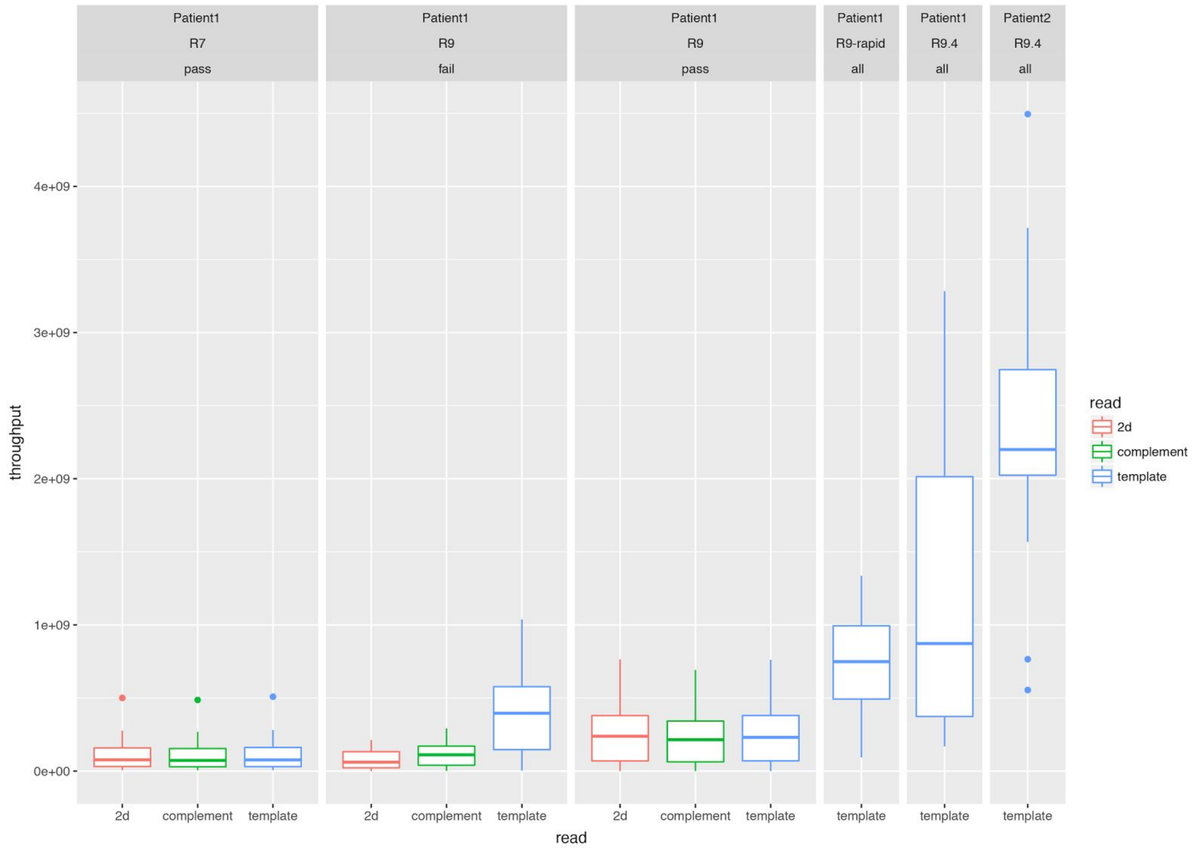

Supplementary Figure 1. Distributions of sequencing throughput for MinION sequencing runs.

For each sequencing run the throughput was calculated as the sum of the read lengths for each type of read (template, complement and 2D). The boxplots indicate the distribution of the throughput for multiple runs, stratified by data type (R7, R9_pass, R9_fail, R9_rapid and R9.4). The y-axis indicates throughput in bases and the x-axis shows read types. R7, R9 and R9.4 represent different nanopore sequencing chemistries for the MinION. Pass and fail indicates reads that were classified as either 'pass' or 'fail' following Metrichor basecalling. 2D indicates consensus reads generated from a template and complement read of a DNA duplex. 1D template and complement indicate reads derived from only one of the two strands (template or complement) of a DNA duplex. 'Rapid' means data from a rapid nanopore library preparation.

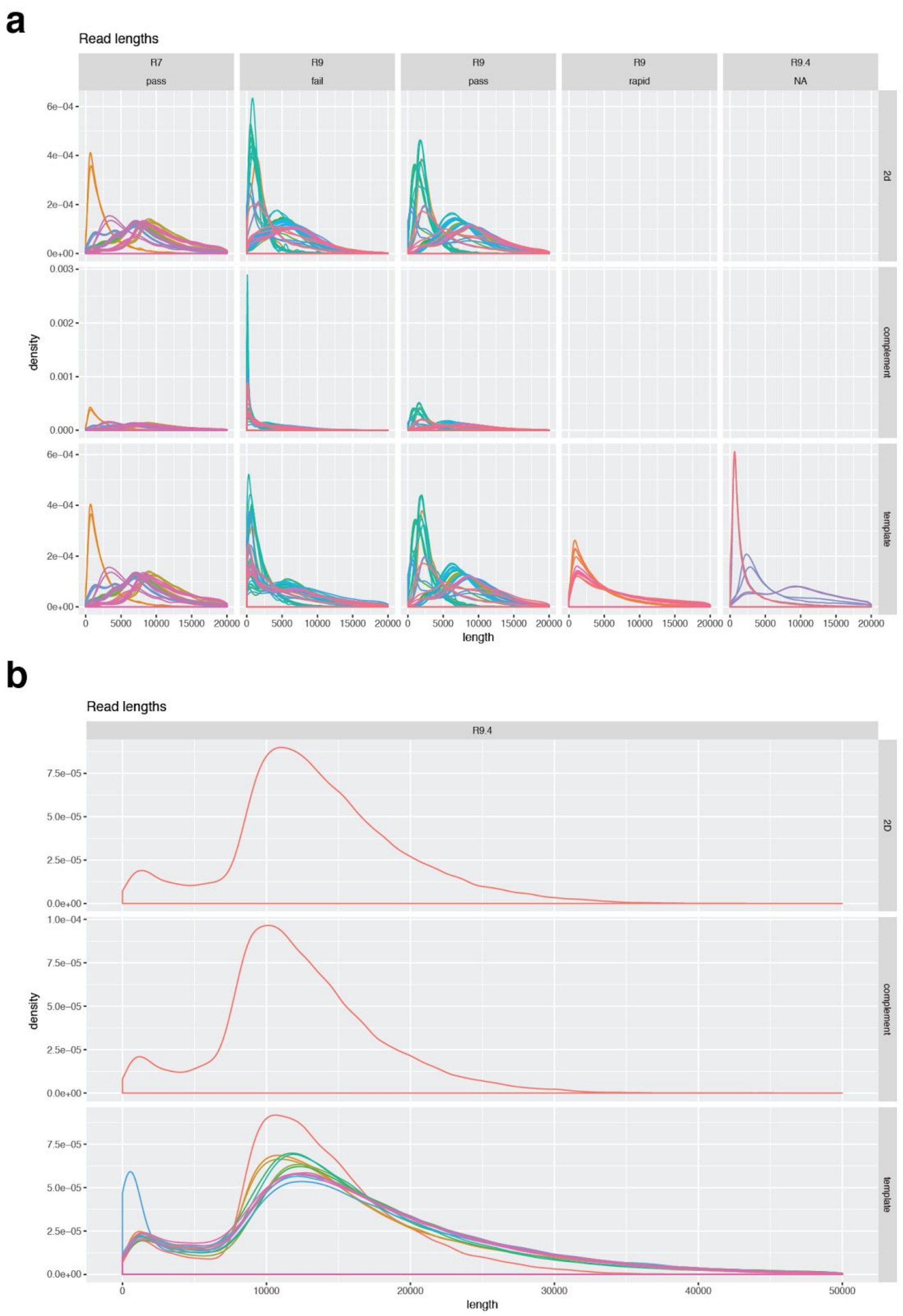

Supplementary Figure 2. MinION run read length distributions.

a Read length distributions for runs for Patient1. **b** Read length distributions for runs for Patient2. MinION sequence runs are indicated by different line colors. Read lengths were calculated for all 1D and 2D reads in the fastq file for each run. Plots are stratified by run type (R7, R9, R9_rapid and R9.4) and data quality ('passed' and 'failed' R9 reads following EPI2ME basecalling) in the horizontal direction and by read type (2D, 1D template, 1D complement) in the vertical direction. R7, R9 and R9.4 represent different nanopore sequencing chemistries for MinION. Pass and fail indicates reads that were classified as either 'pass' or 'fail' following Metrichor basecalling. 2D indicates consensus reads generated from a template and complement read of a DNA duplex. 1D template and complement indicate reads derived from only one of the two strands (template or complement) of a DNA duplex. 'Rapid' means data from a rapid nanopore library preparation.

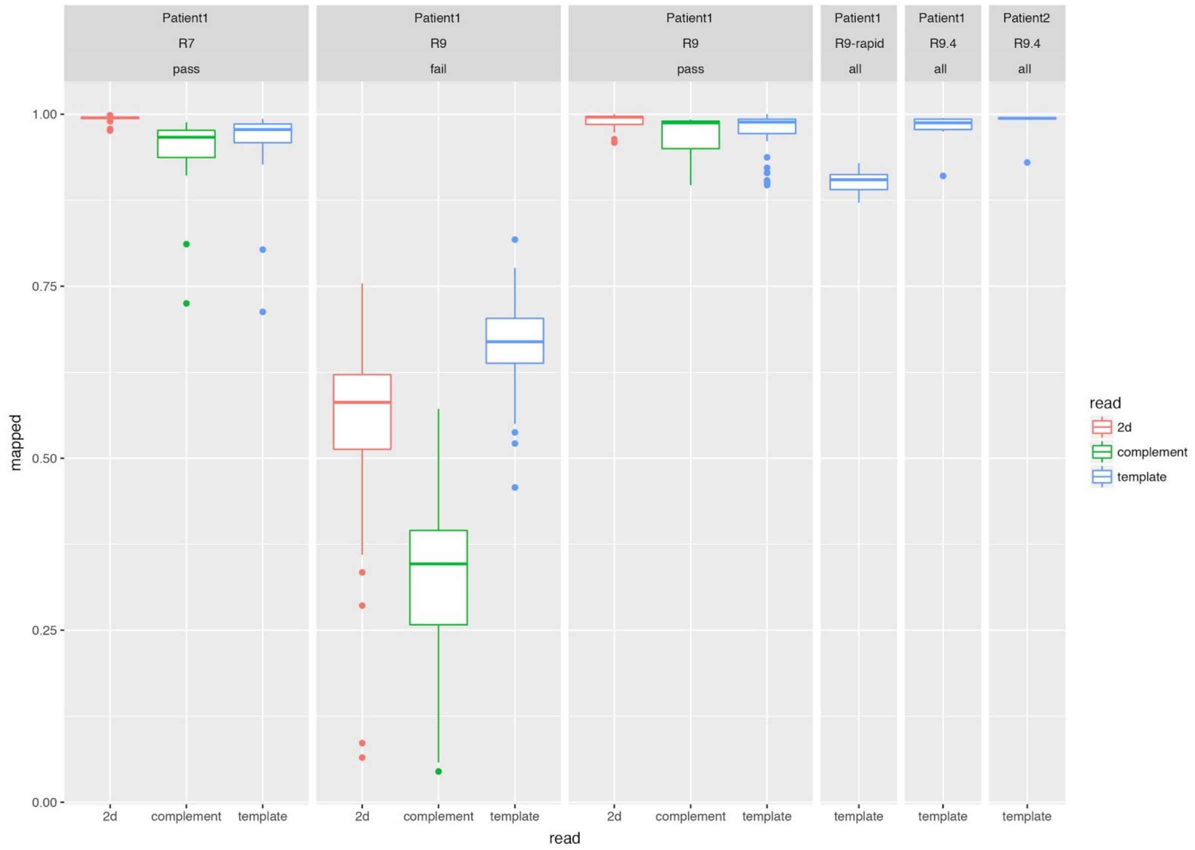

Supplementary Figure 3. Distributions of the percentages of reads mapped by LAST. For each sequencing run the percentage of mapped reads was calculated. The boxplots indicate the distribution of the percentages of mapped reads for multiple runs, stratified by data type (R7, R9_pass, R9_fail, R9_rapid and R9.4). R7, R9 and R9.4 represent different nanopore sequencing chemistries for MinION. Pass and fail indicates reads that were classified as either 'pass' or 'fail' following Metrichor basecalling. 2D indicates consensus reads generated from a template and complement read of a DNA duplex. 1D template and complement indicate reads derived from only one of the two strands (template or complement) of a DNA duplex. 'Rapid' means data from a rapid nanopore library preparation.

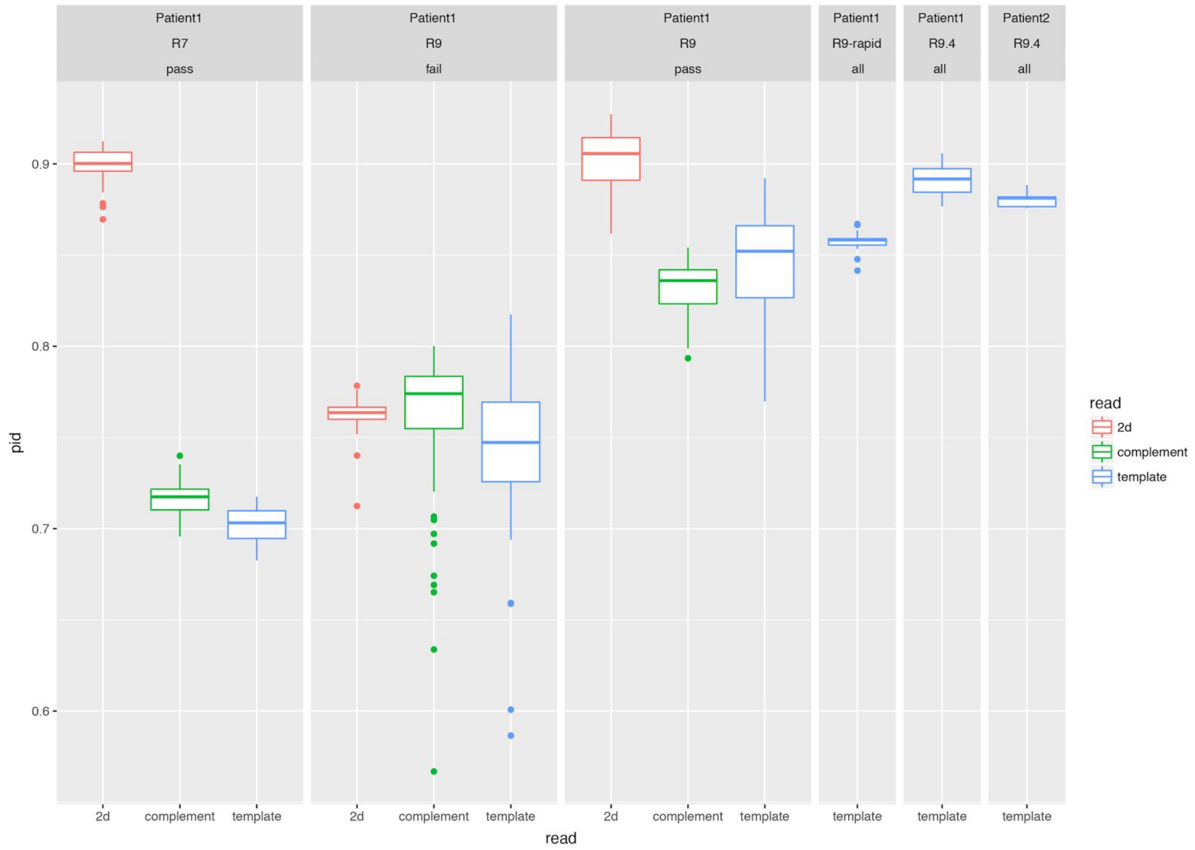

Supplementary Figure 4. Distributions of alignment accuracies of LAST alignments. For each sequencing run the percentage of identical bases (PID) between reference and read sequences were calculated in the alignments. The calculation was done per mapped segment by dividing the amount of identical bases by the length of the mapped segment. Boxplots show the distribution of percentages stratified by run type (R7, R9, R9_rapid and R9.4) and data quality ('passed' and 'failed' R9 reads following EPI2ME basecalling) and by read type (2D, 1D template, 1D complement). R7, R9 and R9.4 represent different nanopore sequencing chemistries for MinION. Pass and fail indicates reads that were classified as either 'pass' or 'fail' following Metrichor basecalling. 2D indicates consensus reads generated from a template and complement read of a DNA duplex. 1D template and complement indicate reads derived from only one of the two strands (template or complement) of a DNA duplex. 'Rapid' means data from a rapid nanopore library preparation.

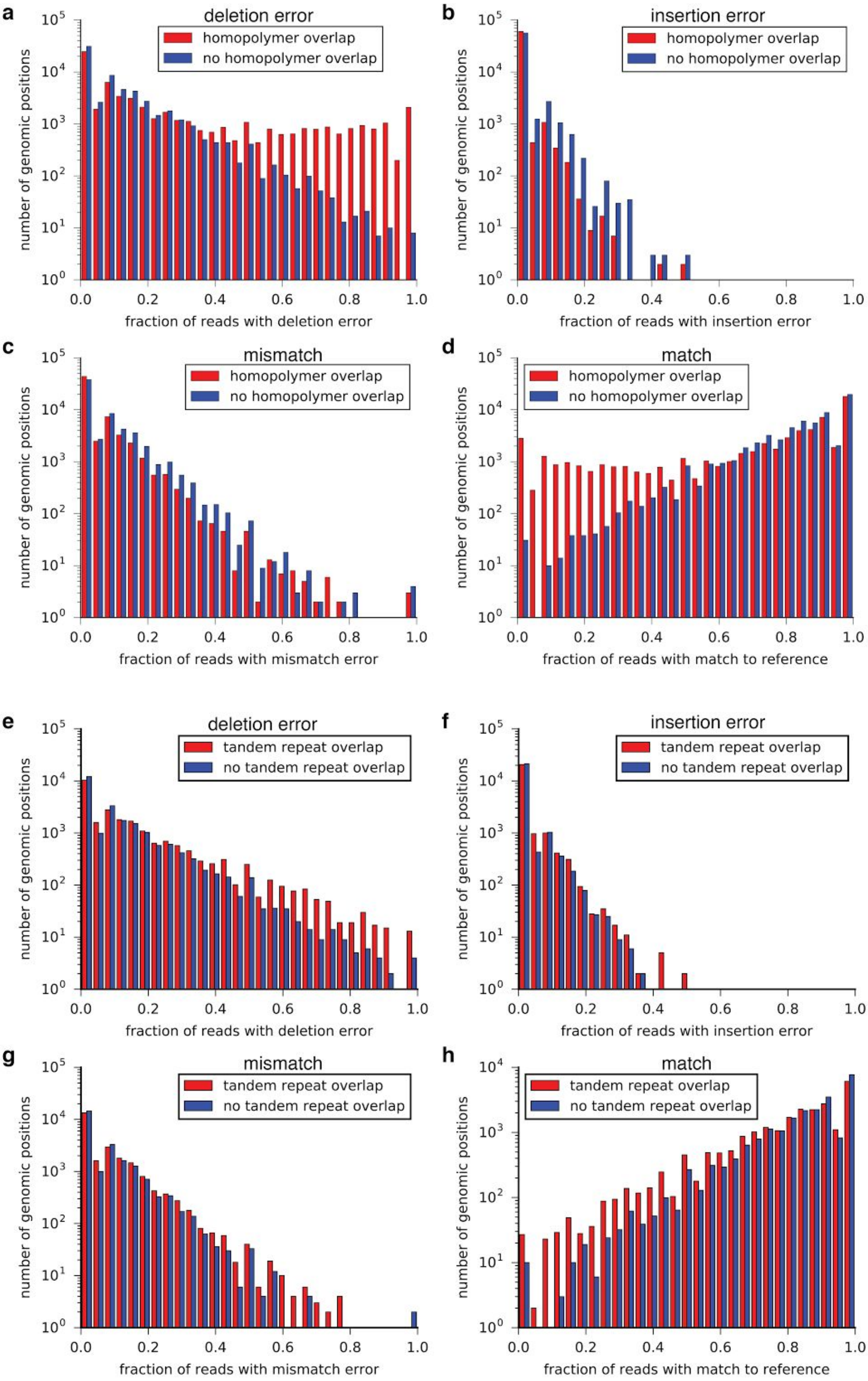
a
deletion error
homopolymer overlap
no homopolymer overlap
number of genomic positions
fraction of reads with deletion error
b
insertion error
homopolymer overlap
no homopolymer overlap
number of genomic positions
fraction of reads with insertion error
c
mismatch
homopolymer overlap
no homopolymer overlap
number of genomic positions
fraction of reads with mismatch error
d
match
homopolymer overlap
no homopolymer overlap
number of genomic positions
fraction of reads with match to reference
e
deletion error
tandem repeat overlap
no tandem repeat overlap
number of genomic positions
fraction of reads with deletion error
f
insertion error
tandem repeat overlap
no tandem repeat overlap
number of genomic positions
fraction of reads with insertion error
g
mismatch
tandem repeat overlap
no tandem repeat overlap
number of genomic positions
fraction of reads with mismatch error
h
match
tandem repeat overlap
no tandem repeat overlap
number of genomic positions
fraction of reads with match to reference

Supplementary Figure 5. Error profiles of R9.4 MinION sequencing data related to homopolymer and tandem repeat context.

A set of 1,064,470 randomly generated genomic positions (excluding polymorphic sites) were sampled from chromosome 1. For each of these positions the fraction of reads with deletion errors, insertion errors and mismatches was determined, based on MinION data from Patient2 (R9.4). In addition, the distance to the closest homopolymer (**Methods**) or tandem repeat (UCSC Simple Repeats track) was calculated. **a-d** Overlap of genomic sites with and without homopolymer overlap (without: >200bp away from nearest homopolymer), stratified by error class (**a** deletion, **b** insertion, **c** mismatch, **d** fraction of bases matching the reference). **e-h** Overlap of genomic sites with and without tandem repeat overlap (with: tandem repeat overlap and no homopolymer overlap, without: >300bp away from nearest tandem repeat and no homopolymer overlap), stratified by error class (**e** deletion, **f** insertion, **g** mismatch, **h** fraction of bases matching the reference).

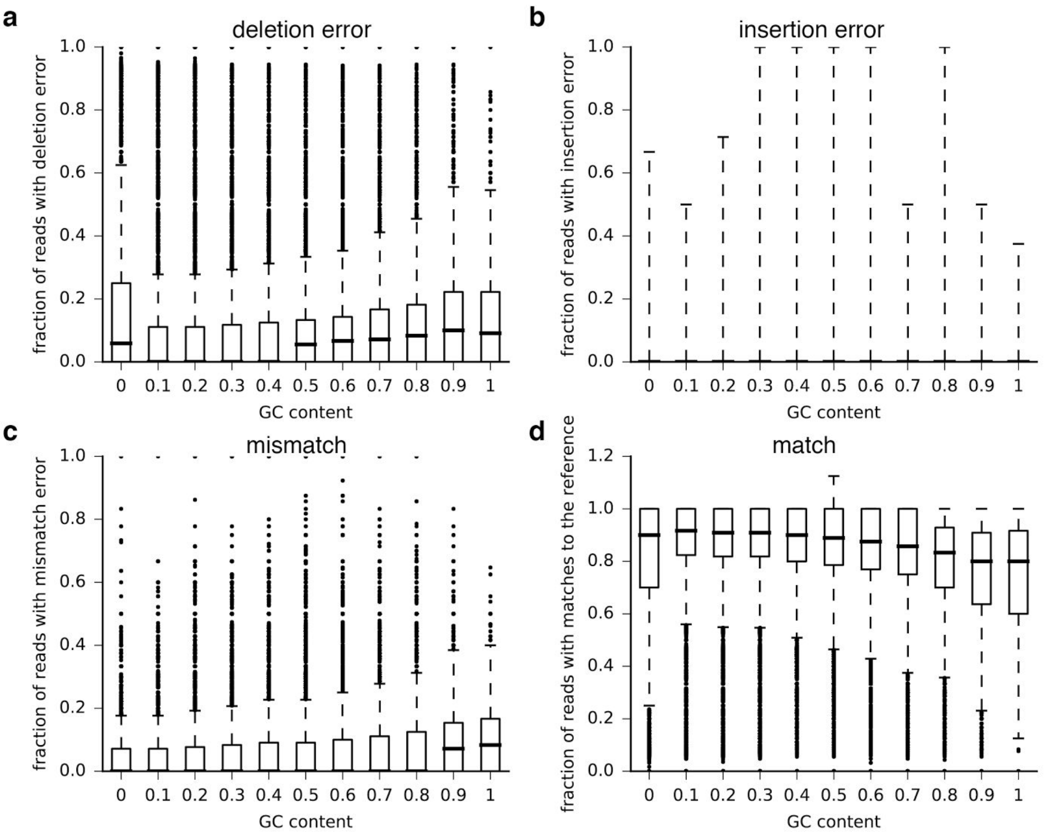

Supplementary Figure 6. Genomic GC content versus error rate in R9.4 MinION sequencing data.

A set of 1,064,470 randomly generated genomic positions (excluding polymorphic sites) were sampled from chromosome 1. For each of these positions the fraction of reads with deletion errors, insertion errors and mismatches was determined, based on MinION data from Patient2 (R9.4). In addition, the GC content of the reference genome was calculated based on a window of 10bp at each examined genomic position. **a** The fraction of deletion errors, **b** insertion errors, **c** mismatches and **d** matches to the reference genome are depicted (y-axis) as a function of genomic GC content (x-axis). For deletion errors, a linear regression model shows a statistically significant dependency of the error rate on the GC content ($p < 10^{-16}$). The estimated coefficient, as change of error fraction per percent of GC-content, is 0.0072 (std. error = 0.0007).

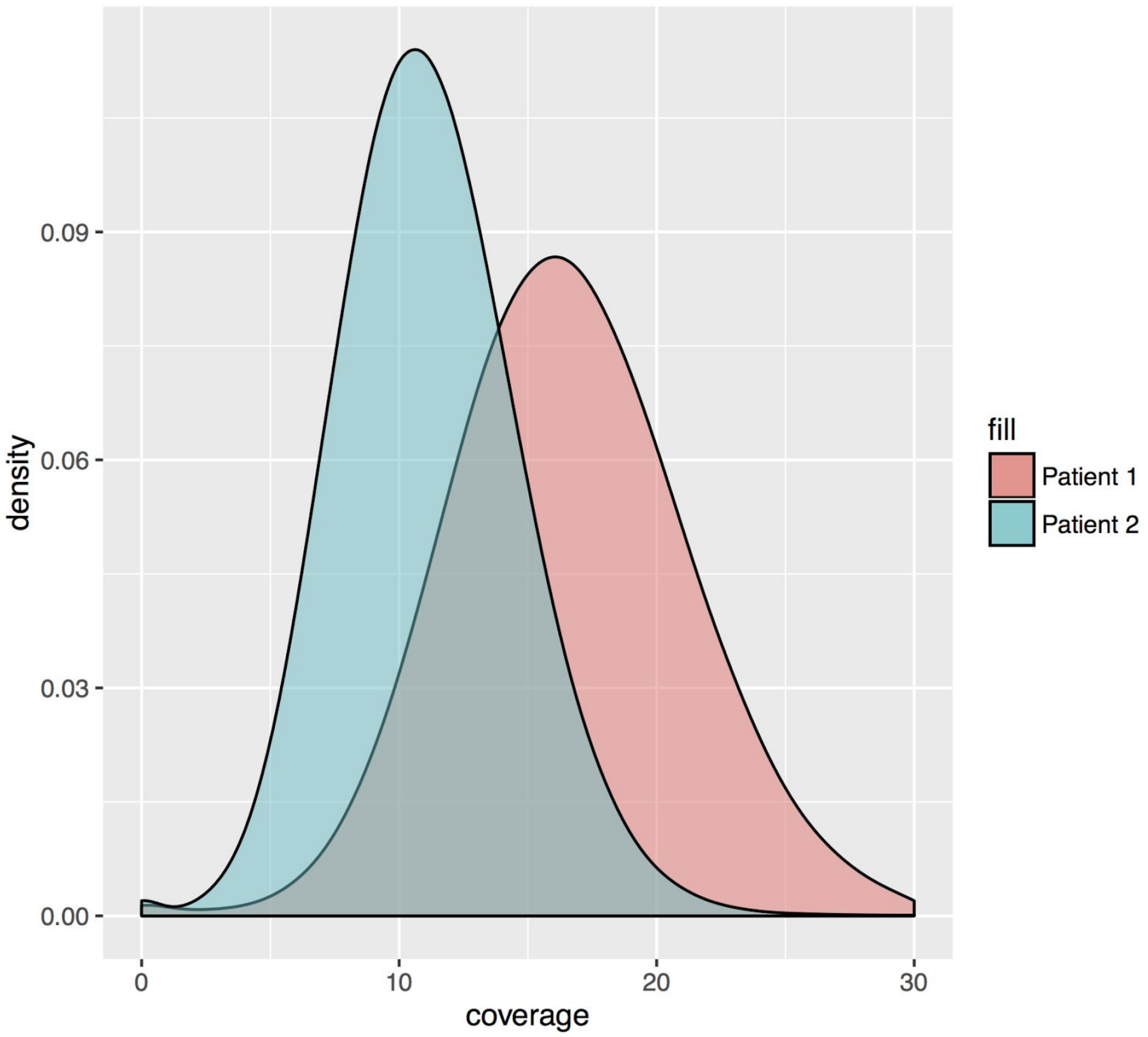

Supplementary Figure 7. Coverage distribution for sequencing data from Patient1 and Patient2.

Coverage distributions were generated by calculating the coverage for 1,000,000 random genomic positions, excluding positions in the gap table downloaded from the UCSC genome browser (GRCh37 gaps in golden path).

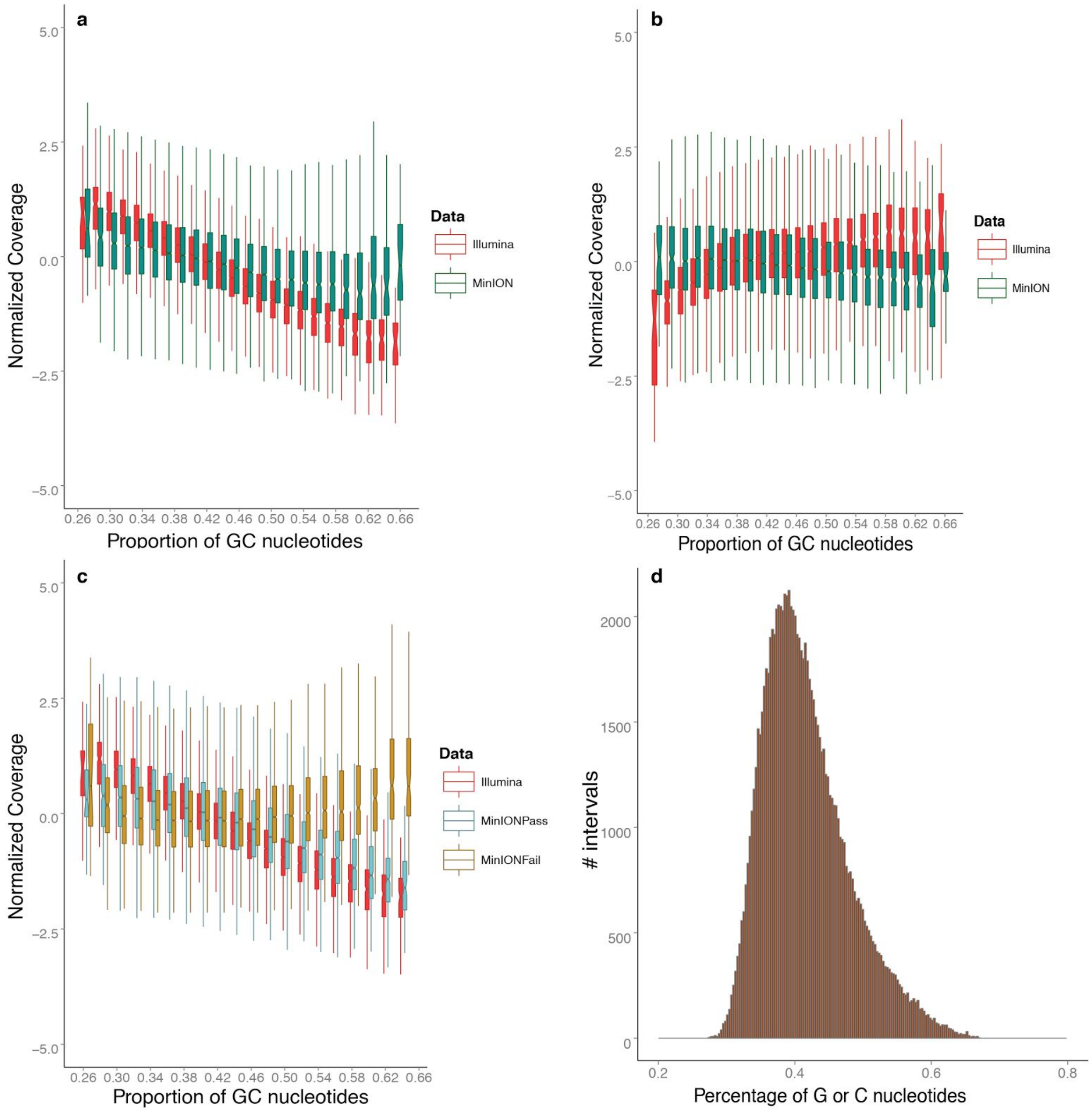

Supplementary Figure 8. Average coverage distribution as a function of GC-content for MinION and Illumina sequencing data of Patient1 and Patient2.

Panels **a** and **b** show statistics of depth of coverage for Illumina data (red) and MinION data (green) for Patient1 and Patient2 respectively. Panel **c** shows statistics of depth of coverage for Illumina data (red), MinION nanopore "pass" data (green) and MinION nanopore "fail" data (dark yellow) of Patient1. Panel **d** shows the GC content distribution across our randomly sampled intervals. The average coverages across 100,000 randomly sampled 5kb genomic intervals were used in each panel. Average coverage outliers, defined as 6 or more interquartile distances away from the median, were discarded for each technology respectively. The remaining data were normalized to N(0,1), to account for different genome-wide sequencing average coverage and binned by GC-content. A linear regression model shows a statistically significant dependency of coverage depth on the GC content expressed as percentage, for both technologies (p < 10^-16). The estimated coefficients, as number of standard deviations of change, per percentage of GC-content, are -0.094 (std.

error = 0.0004) and -0.029 (std. error = 0.0004) for the Illumina and MinION data of Patient1 respectively (panel **a** non-binned data). Conversely the estimated coefficients for Patient2 are 0.033 (std. error = 0.0004) and -0.018 (std. error = 0.0004) for the Illumina and MinION nanopore data respectively (panel **b** non-binned data).

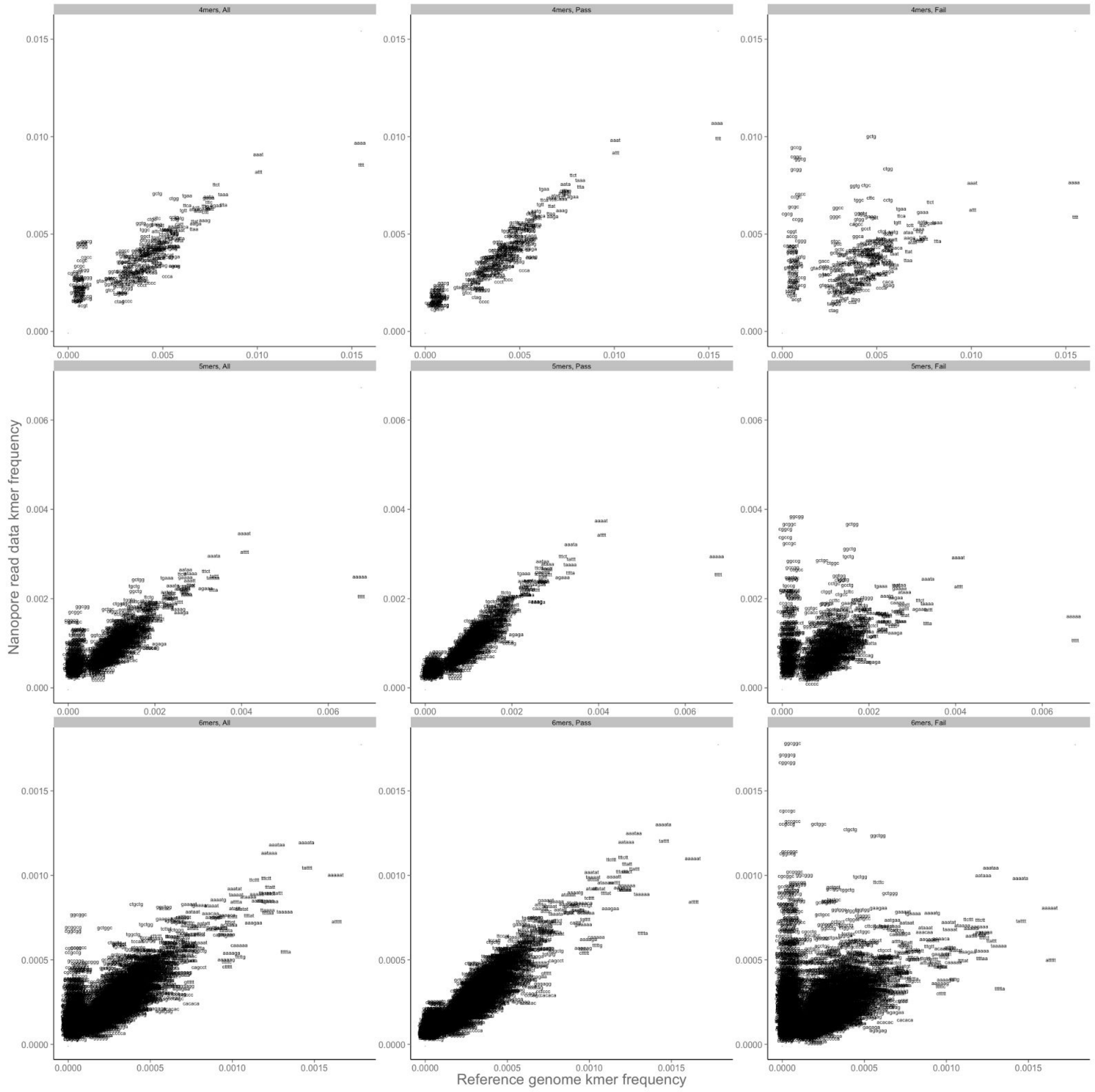

Supplementary Figure 9. K-mer distribution in MinION sequencing data of Patient1. Plotted observed (MinION data) versus expected (GRCh37 reference genome) relative k-mer frequencies for 4-mers (**top**), 5-mers (**middle**) and 6-mers (**bottom**). The expected kmer frequencies are computed from the relative frequency of each kmer on the reference genome primary assembly for each k-mer size. The MinION data k-mer frequency was similarly computed, across all MinION reads, further stratified by “pass” (**middle**) or “fail” (**right**) read status. The “All” (**left**) represents the aggregate “pass” and “fail” MinION data.

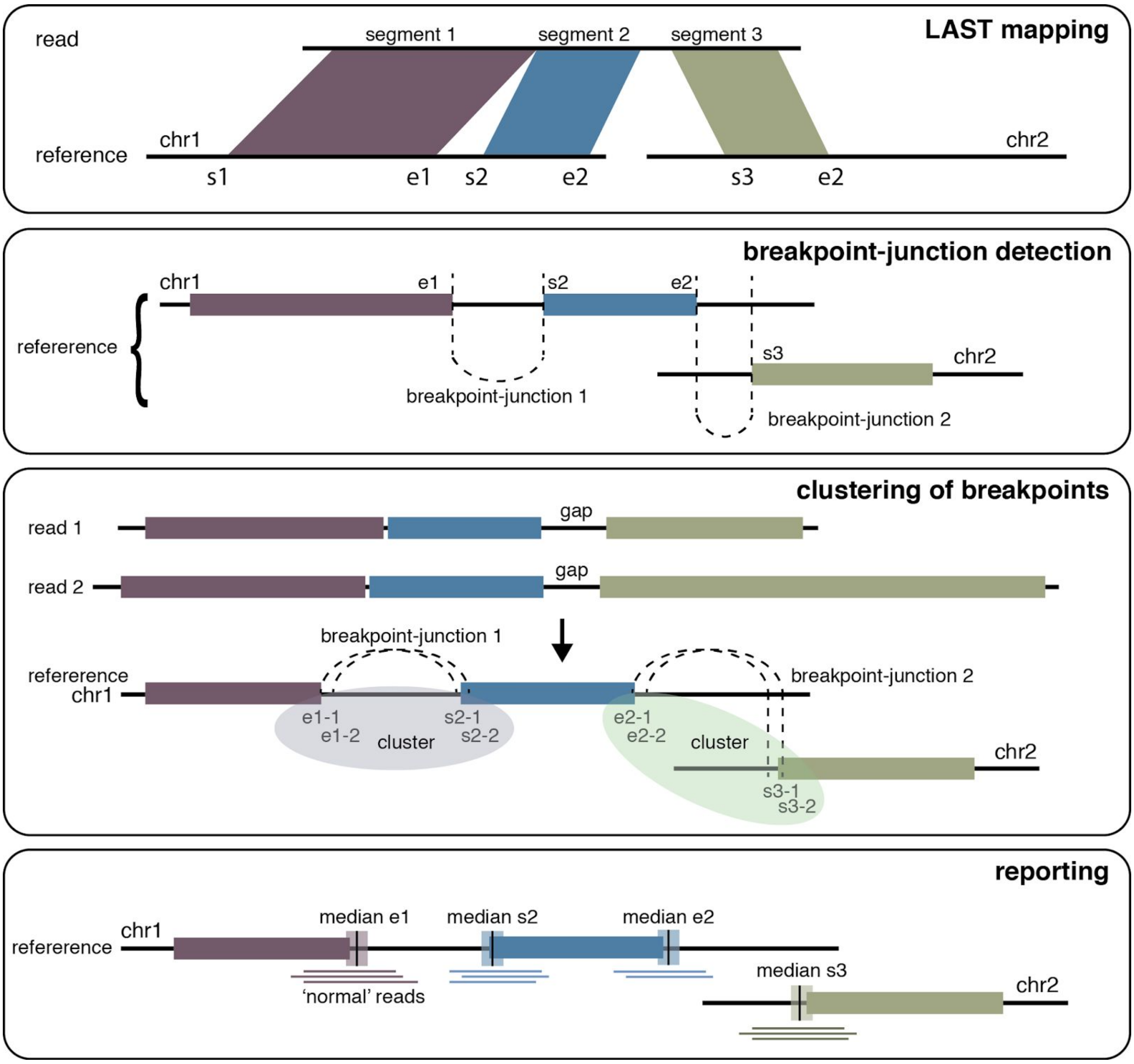

Supplementary Figure 10. Overview of NanoSV algorithm.

NanoSV uses LAST mapping output for discovery of SVs. In a first step candidate breakpoint junctions are detected using split read mappings. Candidate breakpoint junctions are subsequently clustered across multiple reads based on the overlap of junction coordinates and orientation. Clusters of breakpoint junctions are reported as SVs in VCF format. The tool is available on github: https://github.com/mroosmalen/nanosv.

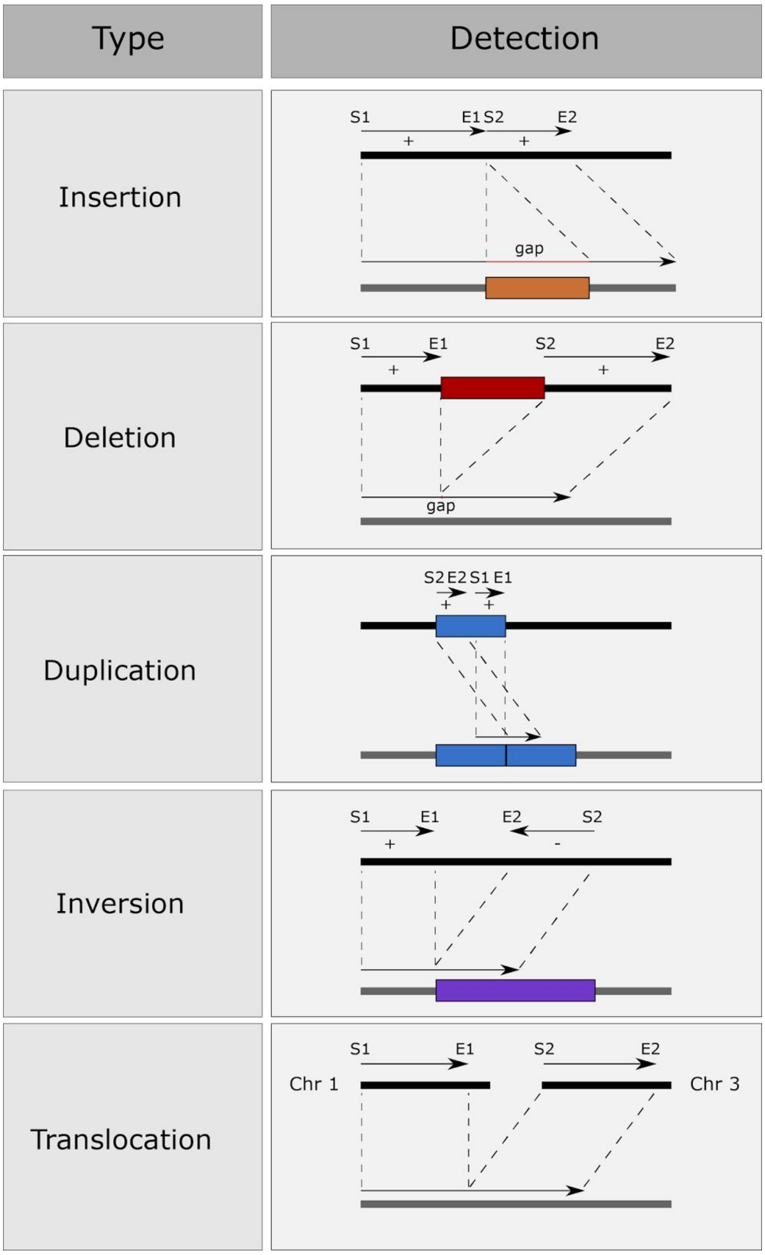

Supplementary Figure 11. Detection of different SV types by NanoSV.
NanoSV detects most types of breakpoints junctions with the exception of insertions consisting of unmapped repeat elements which are longer than the nanopore read lengths, e.g. LINE insertions may be missed if the read length falls below the typical length of LINE elements (~6kb). Genomic coordinates of mapped segments are indicated by *s1*/*s2* (start of segments) and *e1*/*e2* (end of segments). Gaps within reads represent unmapped segments, which may result from repeat insertions or complex variations. Deletions are discerned from insertions if the gap length is smaller than the distance between the joined genomic positions (*s2*-*e1* which represents SV size for variants other than insertions).

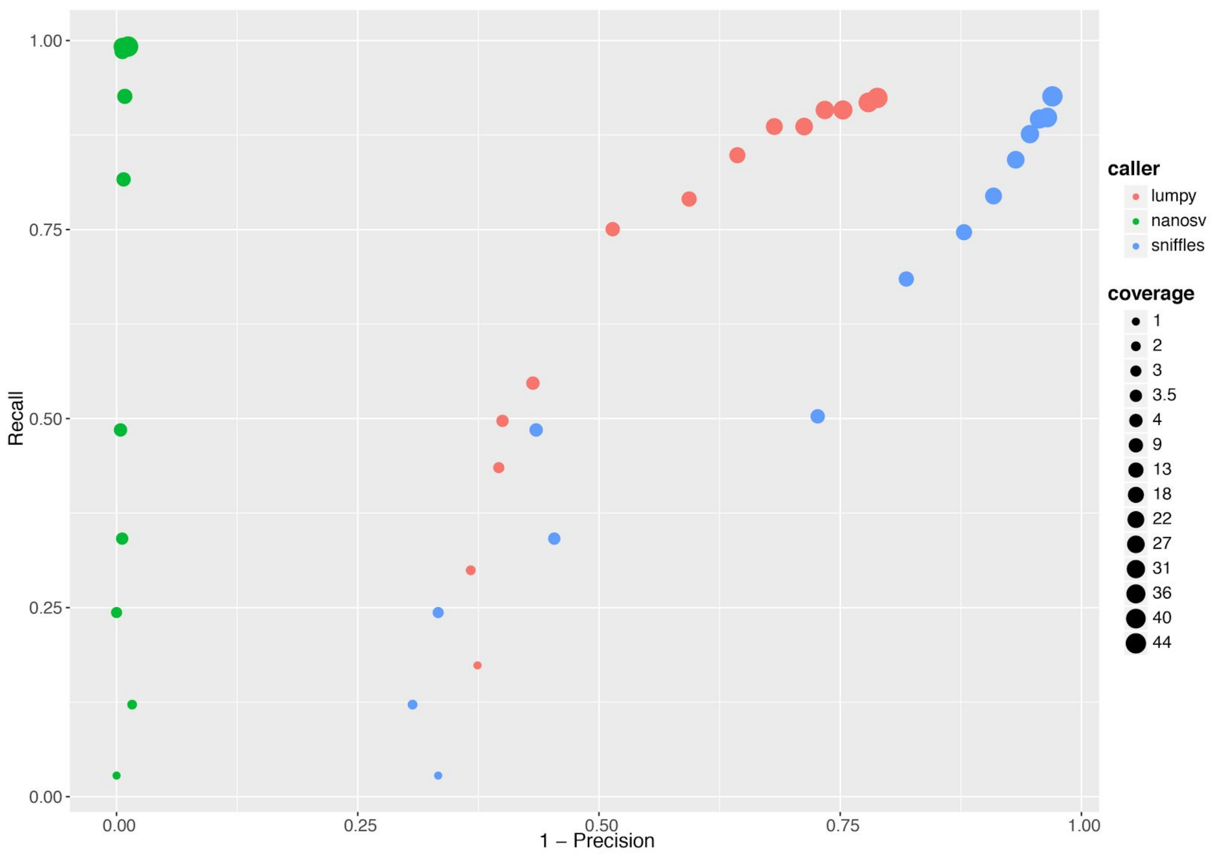

Supplementary Figure 12. Recall-precision curve for SV calling performance on simulated nanopore data.

Breakpoints (501) were simulated on reference chr1 and based on the resulting chromosomal sequence nanopore reads were simulated using NanoSim[1]. SV calling using Lumpy[2], Sniffles[3] and NanoSV was performed on subsets of the simulated nanopore reads to estimate the effect of read coverage. The recall (true positives/true positives + false negatives) and precision (true positives/true positives + false positives) was calculated for each call set, without any additional post-calling filters being applied.

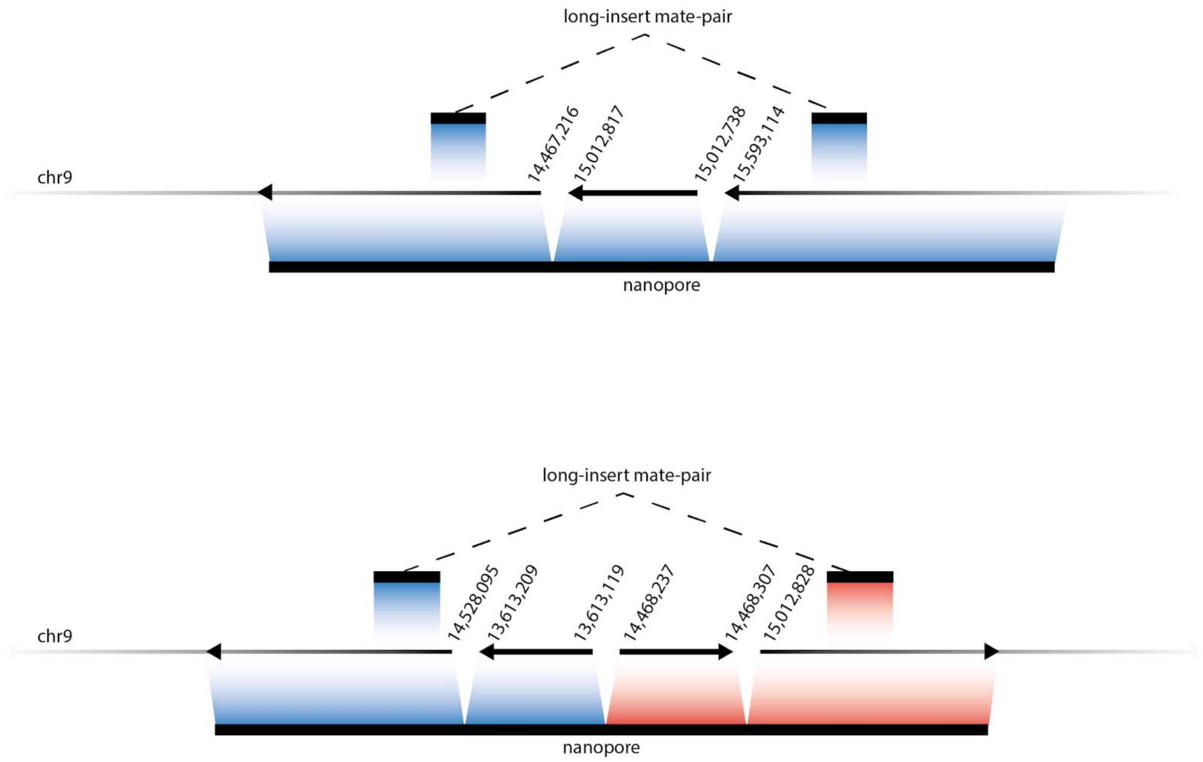

Supplementary Figure 13. Structure of two complex breakpoint-junctions in Patient2 chromothripsis.

Long-insert mate-pair sequencing was previously used to study the chromothripsis in Patient2[4]. The long-insert size of these mate-pair libraries hampers detection of short chromosomal segments, because the short sequence reads can jump over the short segments and only reveal the connection between the segments flanking these short segments. In the upper panel, an 80bp segment from chr9 is depicted, which was identified using nanopore reads and confirmed by Sanger sequencing. The lower panel highlights two adjacent short genomic segments - both from chr9 - that were missed by the long-insert mate-pair sequencing, but detected by nanopore reads and subsequent PCR and Sanger sequencing.

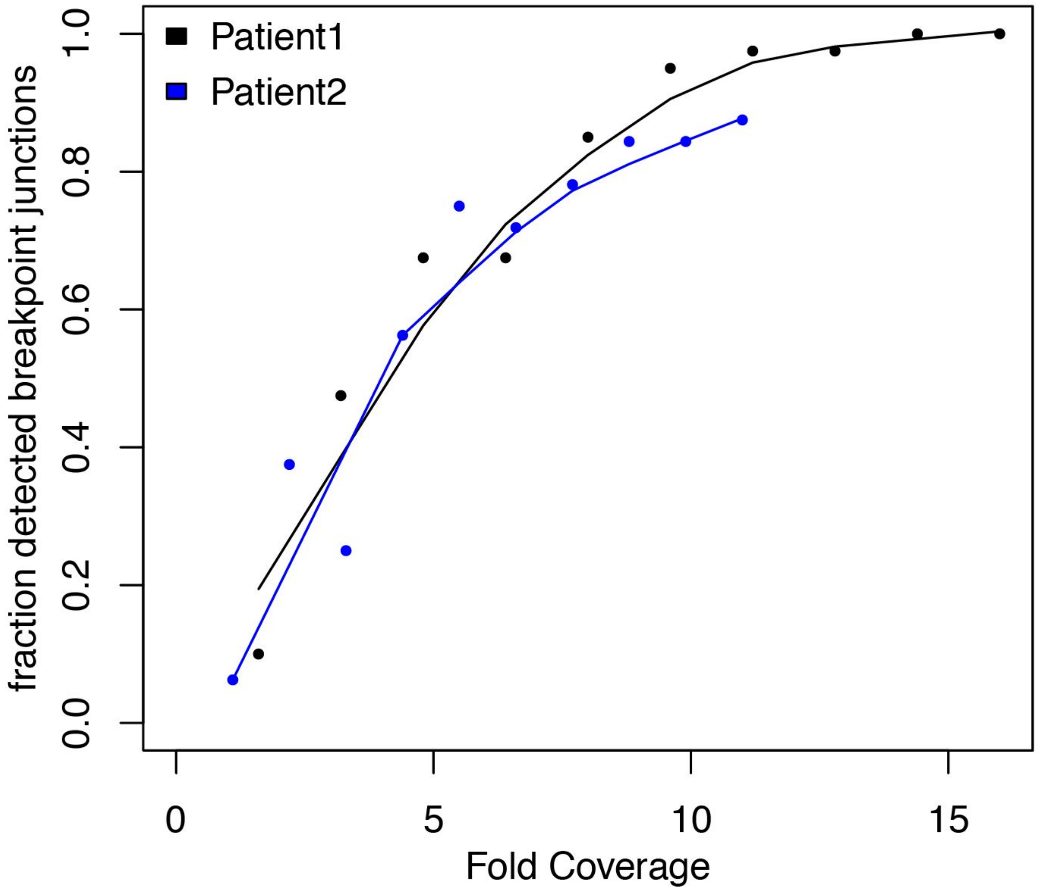

Supplementary Figure 14. The effect of subsampling the MinION sequencing data on chromothripsis breakpoint-junction detection.

Nanopore sequencing reads were subsampled from 10% to 90% of the original data and each subsampled dataset was analyzed using NanoSV to determine the fraction of known chromothriptic breakpoint-junctions that could be detected. Below a coverage of ~14x (Patient1), the fraction of detected breakpoint junctions drops below 1.

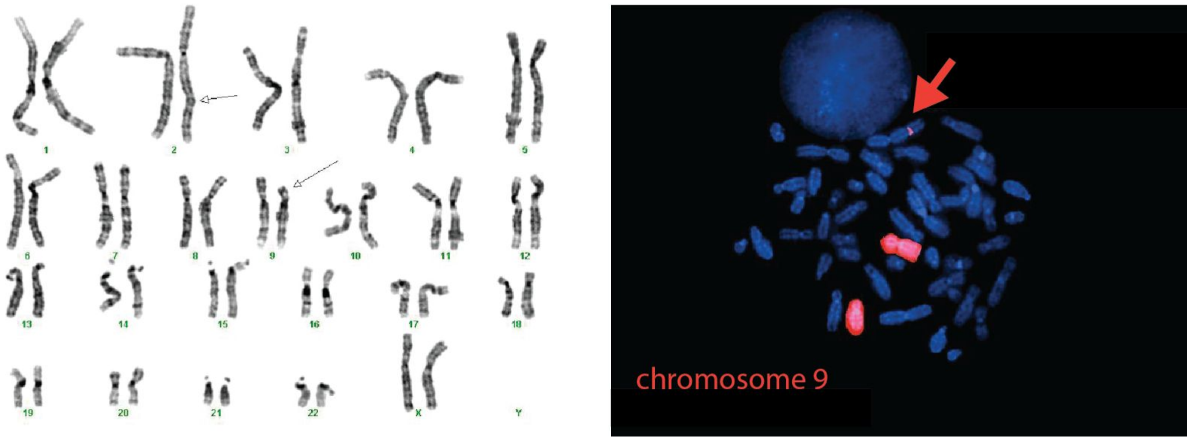

Supplementary Figure 15. Karyotype and chromosome 9 painting derived from Patient1 chromosome spreads.

Left panel shows the patient karyotype. Arrows indicate chromosome 2 and chromosome 9. The right panel displays a chromosome 9 paint (red) demonstrating an insertion of a part of chromosome 9 into chromosome 2 (arrow).

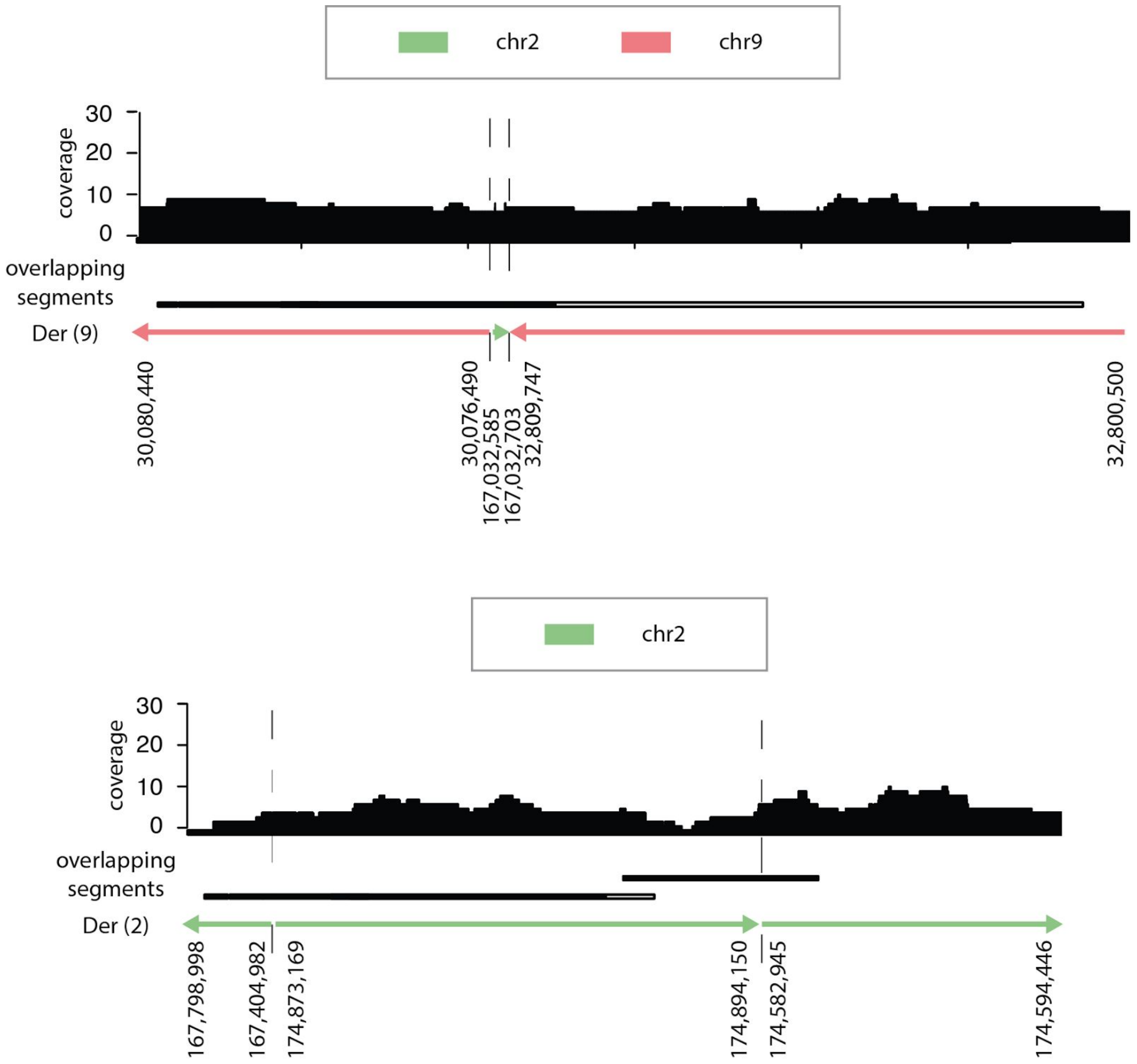

Supplementary Figure 16. Reference-assisted assembly of chromothripsis regions in Patient1.

Order and orientation of chromosomal regions involved in the chromothripsis rearrangements of Patient1 is depicted by colored lines with arrowheads. The resulting chromosomal configuration is based on overlapping nanopore reads derived from the paternal haplotype of Patient1. Nanopore reads that are instrumental for segment connectivity are indicated by black bars. The coverage track has been generated from all paternal reads mapping to the respective chromosomal segments. The order and orientation of the joined chromosomal segments matches the chromothripsis structure that is described in **Figure 3**.

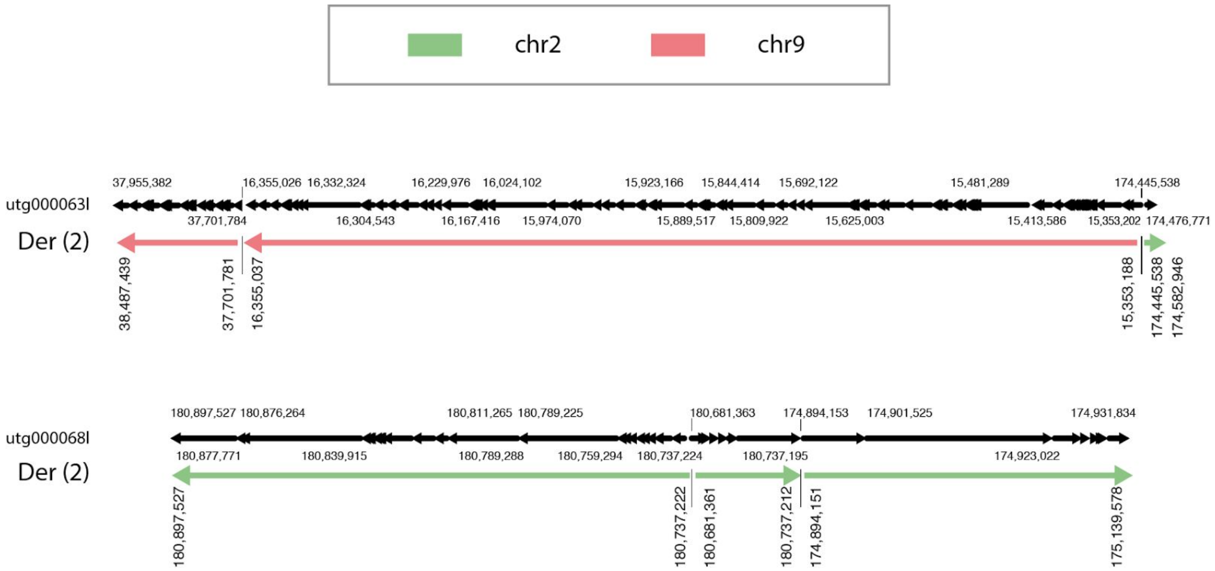

Supplementary Figure 17. Contig structure produced by Miniasm assembly of chromothripsis regions in Patient1.

Order and orientation of chromosomal regions involved in the chromothripsis rearrangements of Patient1 is depicted by colored lines with arrowheads and was obtained as for **Figure 3**. The structure for two chromothriptic regions, containing three genomic segments each, was supported by contiguous sequences (contigs) resulting from Miniasm[5] assembly of nanopore reads, excluding reads that were assigned to the maternal haplotype. Both contigs (utg000068I and utg000063I) support part of the structure of derivative chromosome 2. The black arrows indicate the positions and orientations of contig segments mapped to the human reference genome (GRCh37).

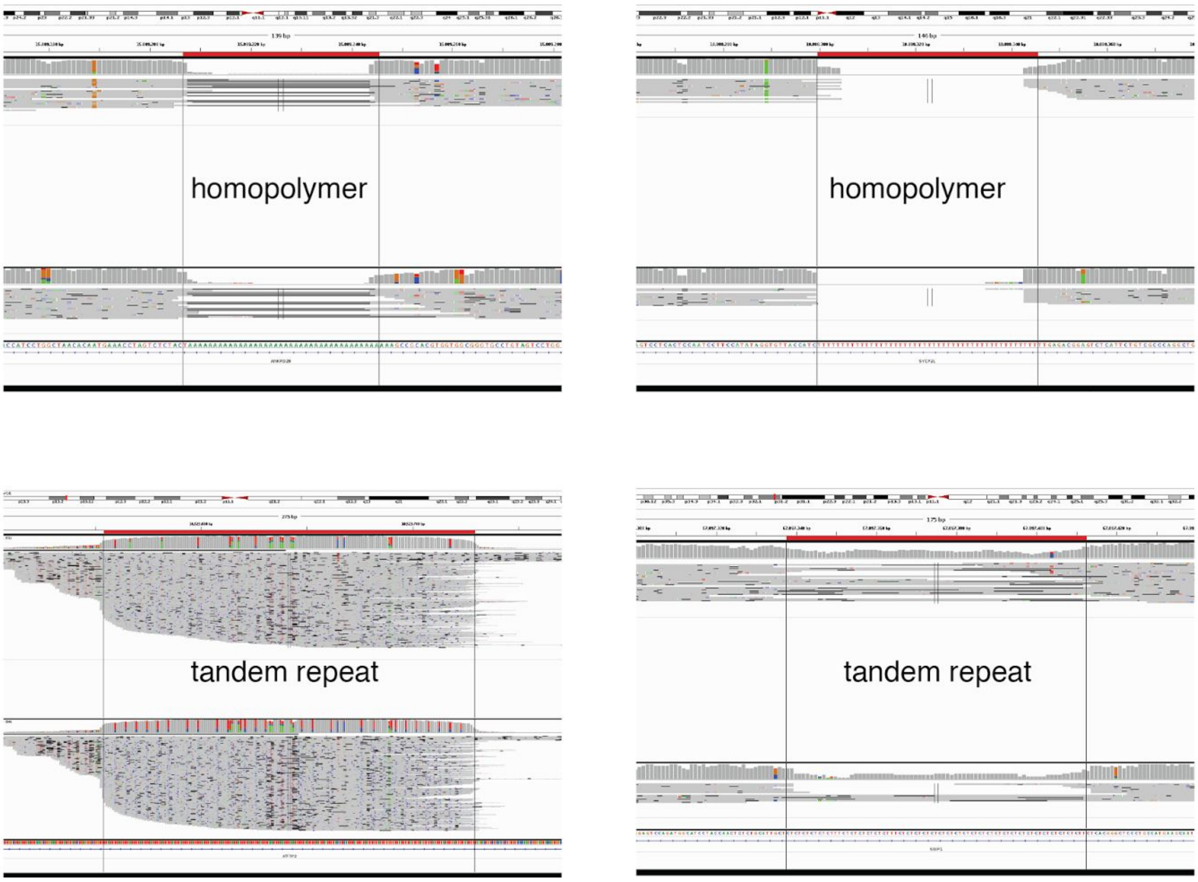

Supplementary Figure 18. IGV screenshots showing MinION nanopore read alignments in homopolymer and tandem repeat regions.

For each panel the upper alignments are from Patient1 and the lower alignments are from Patient2 MinION read data. The lower left panel represents a NanoSV predicted duplication call and the remaining three examples represent NanoSV predicted deletions.

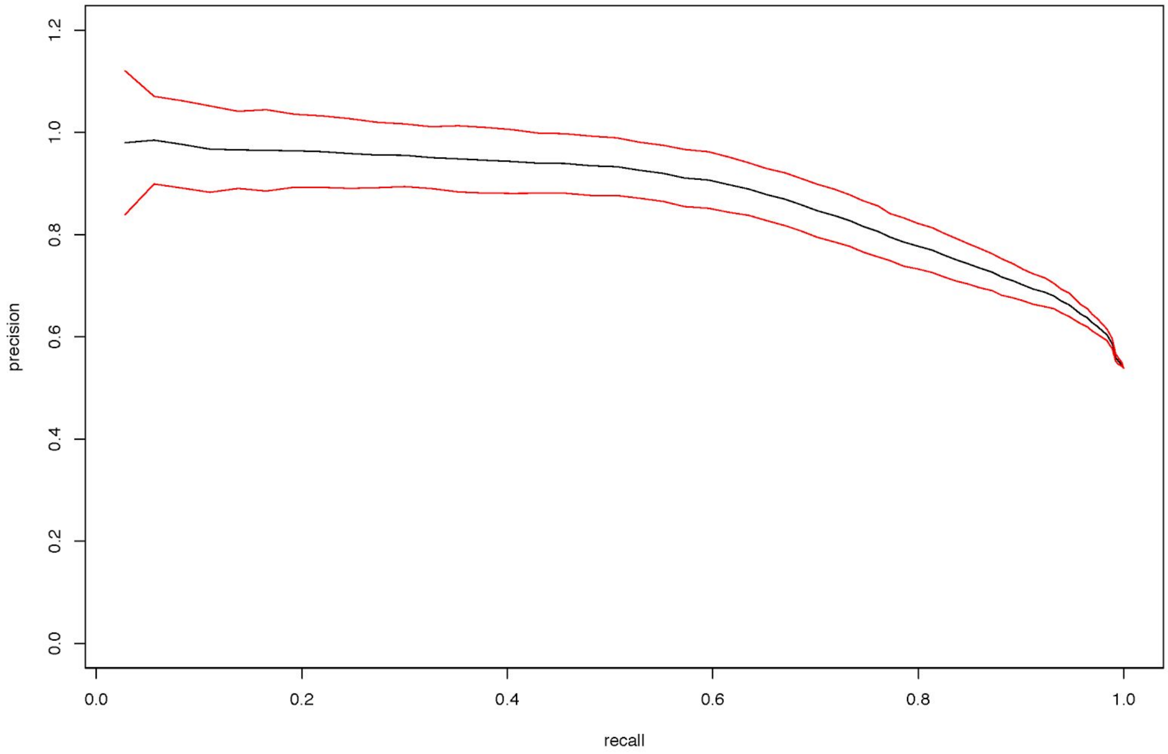

Supplementary Figure 19. Recall-precision curve obtained from training and cross-validation on NanoSV SV calls.

The illustrated ROC curve is obtained from 100 cross-validation random forest training runs (split 90%-10% for training-testing) from the total set of 354 true positive and 300 true negative SVs from the NA12878 sample. The chosen, optimal operating point has a precision of 82% at a recall rate of 75%.

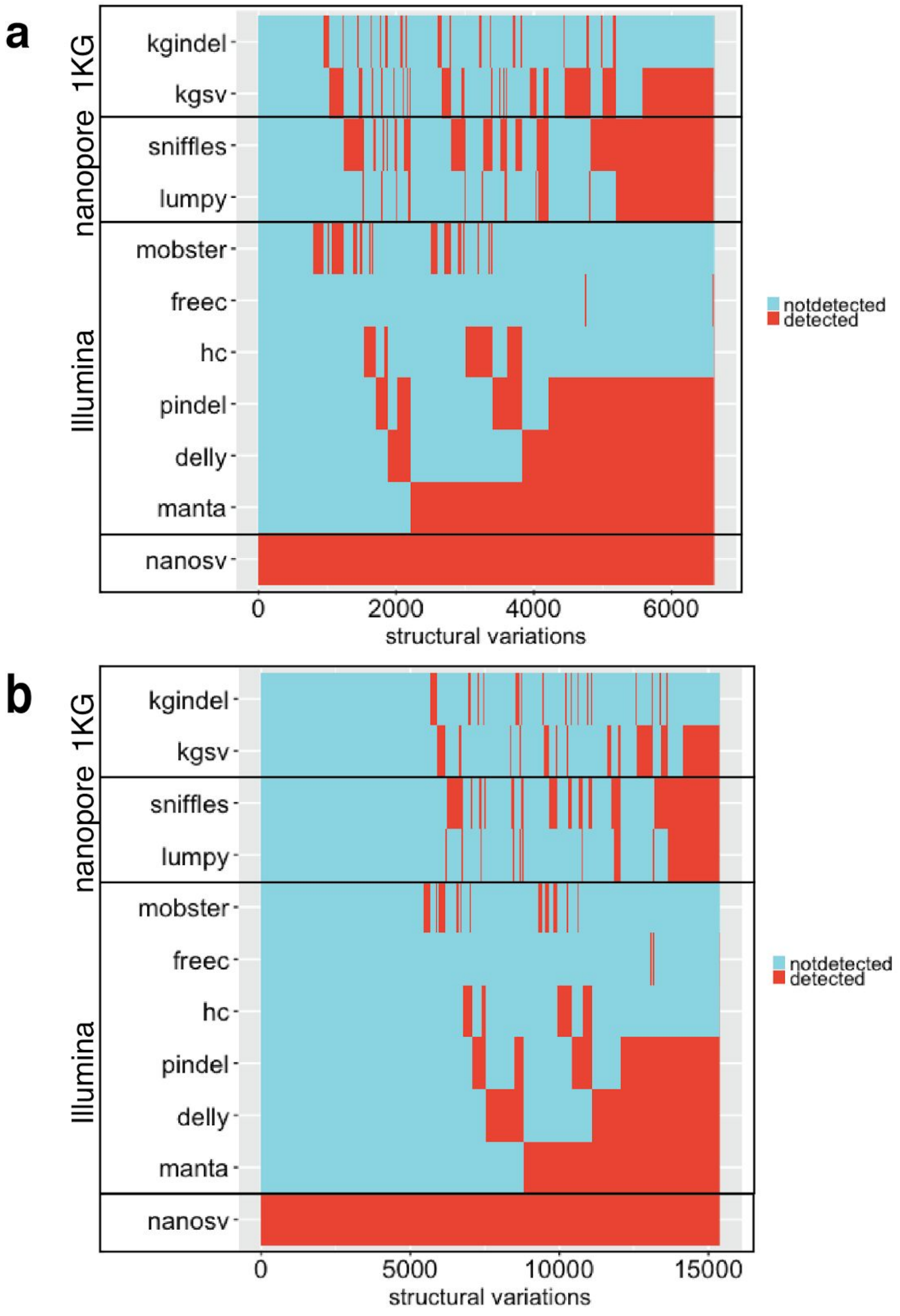

Supplementary Figure 20. Heatmap showing the overlap of SV calls between different callers and SV datasets.

We used the NanoSV SV call set of Patient1 and Patient2 as a basis for intersection with SV call sets generated from Illumina data, using six different tools. Additionally, we used two tools for detection of SVs in the Nanopore data from Patient1 and Patient2. Finally, we intersected the NanoSV calls with the 1000 Genomes phase 3 consensus calls[6]. **a** Heatmap showing overlaps of 6,616 NanoSV SVs predicted as true positive by a random forest classifier (**Methods**). **b** Heatmap showing overlaps of the initial call set consisting of 15,369 candidate NanoSV SVs, following filtering for SVs that overlap homopolymers and tandem repeats (**Methods**).

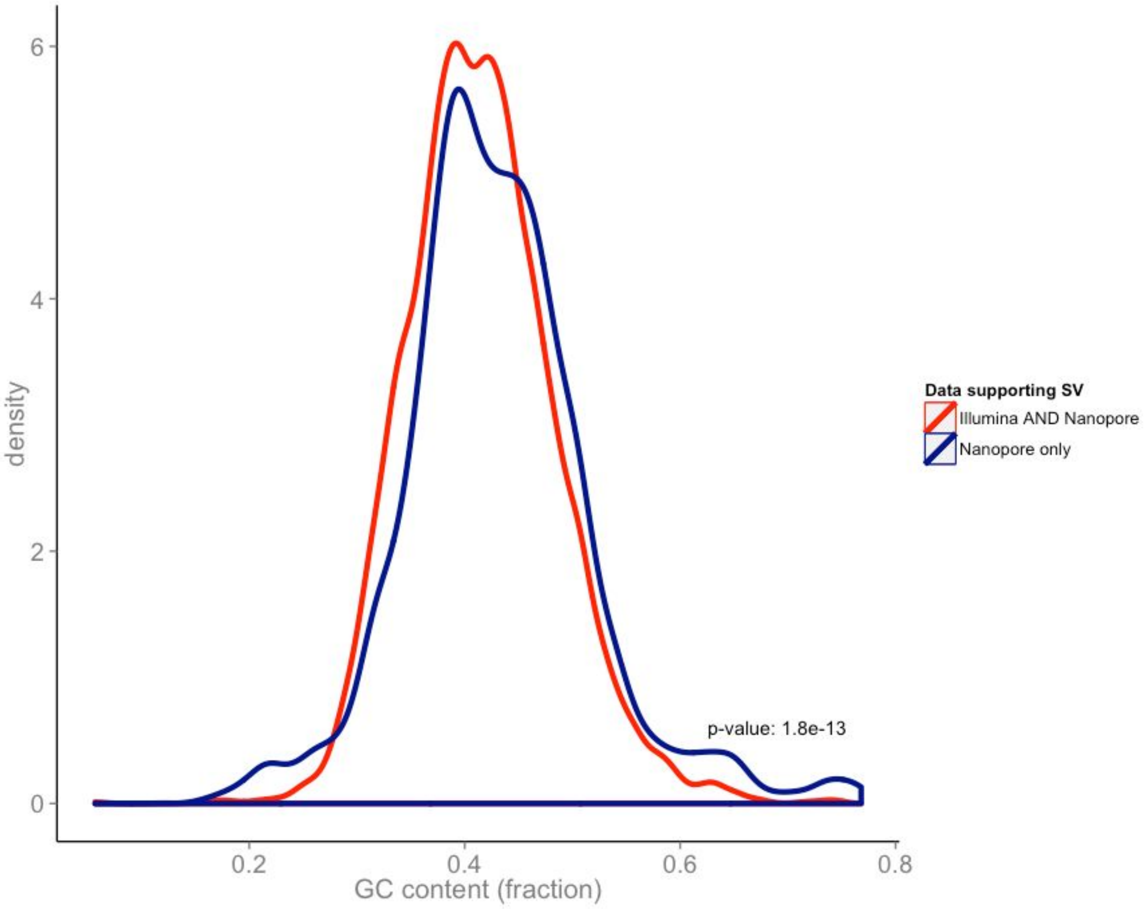

Supplementary Figure 21. GC bias of nanopore specific SVs.
GC content distributions across 500 base-pair windows around the high confidence set of SV calls that are detected in both Illumina and MinION nanopore data (red) and nanopore data only (blue). The average GC content in the regions where an SV is detected only in the nanopore data is 1.4% higher than the the average GC content where an SV is detected in both Illumina and MinION nanopore data (Welch two sample t-test: p-value = 1.8e-13, 95% CI = 1.0 - 1.8).

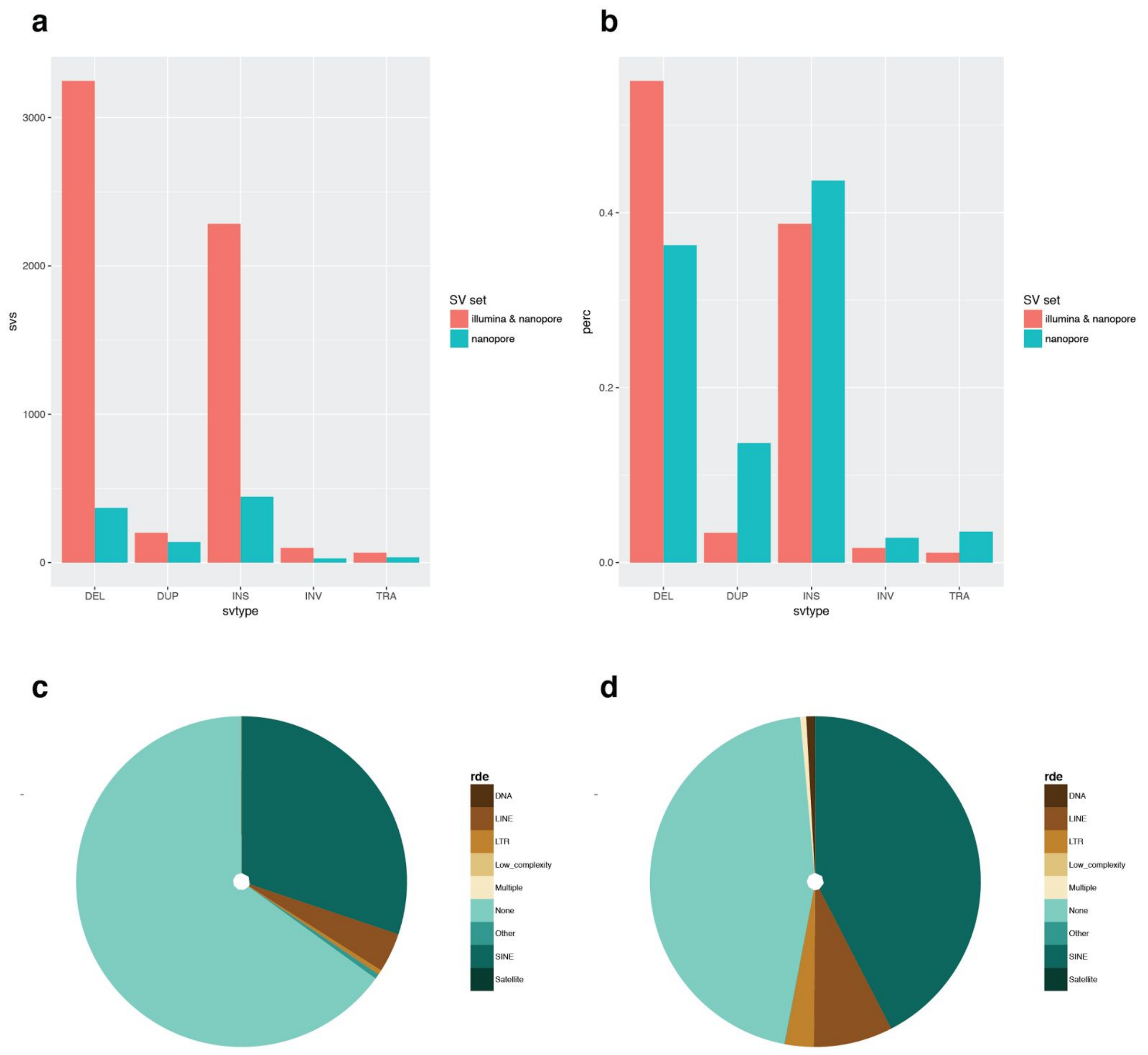

Supplementary Figure 22. Patient1 and Patient2 cumulative distributions of SVs.
We plotted numbers of SV calls across SV types (**a** and **b**) and across SV annotations (**c** and **d**), after random forest filtering. **a** shows the histogram of SV type across both patients, subsetted for the "Illumina and nanopore" data and "nanopore" only data. **b** shows the SV type distribution for the same subsets as **a**. **c** shows the annotations distribution, by class, for all deletions detected in both nanopore and Illumina data. **d** shows the annotations distribution, by class, for all insertions detected in both nanopore and Illumina data.

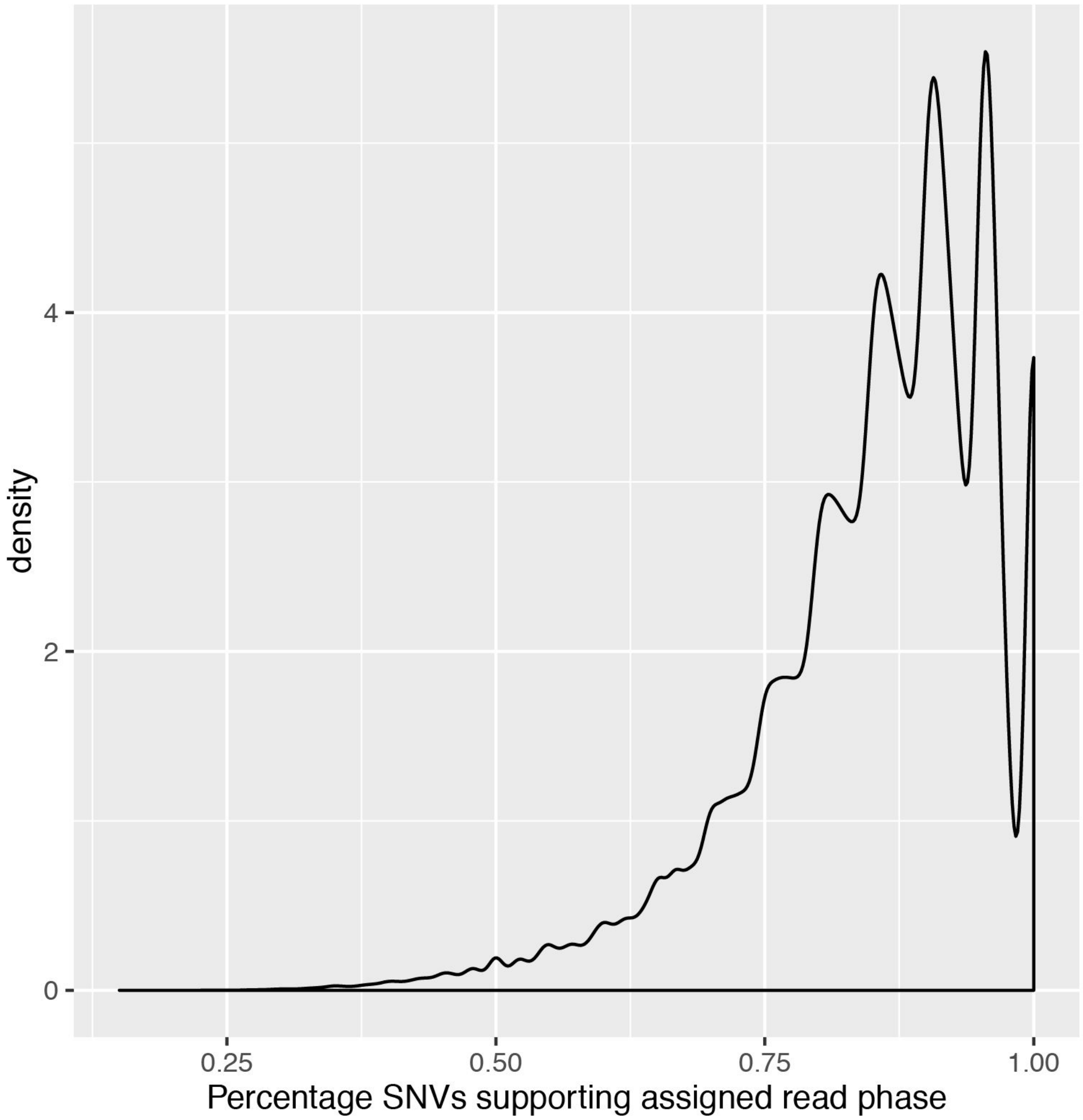

Supplementary Figure 23. Nanopore read phase support.

The plot show the distribution (density) of the percentage *p* of SNVs per read supporting the read phase of each nanopore read covering at least 20 phase-informative SNVs. The percentage *p* is defined as SNV_{supp}/SNV_{total}, where SNV_{supp} is the number of phase-informative SNVs that support the read phase and SNV_{total} is the total number of phase-informative SNVs covered by the nanopore read.

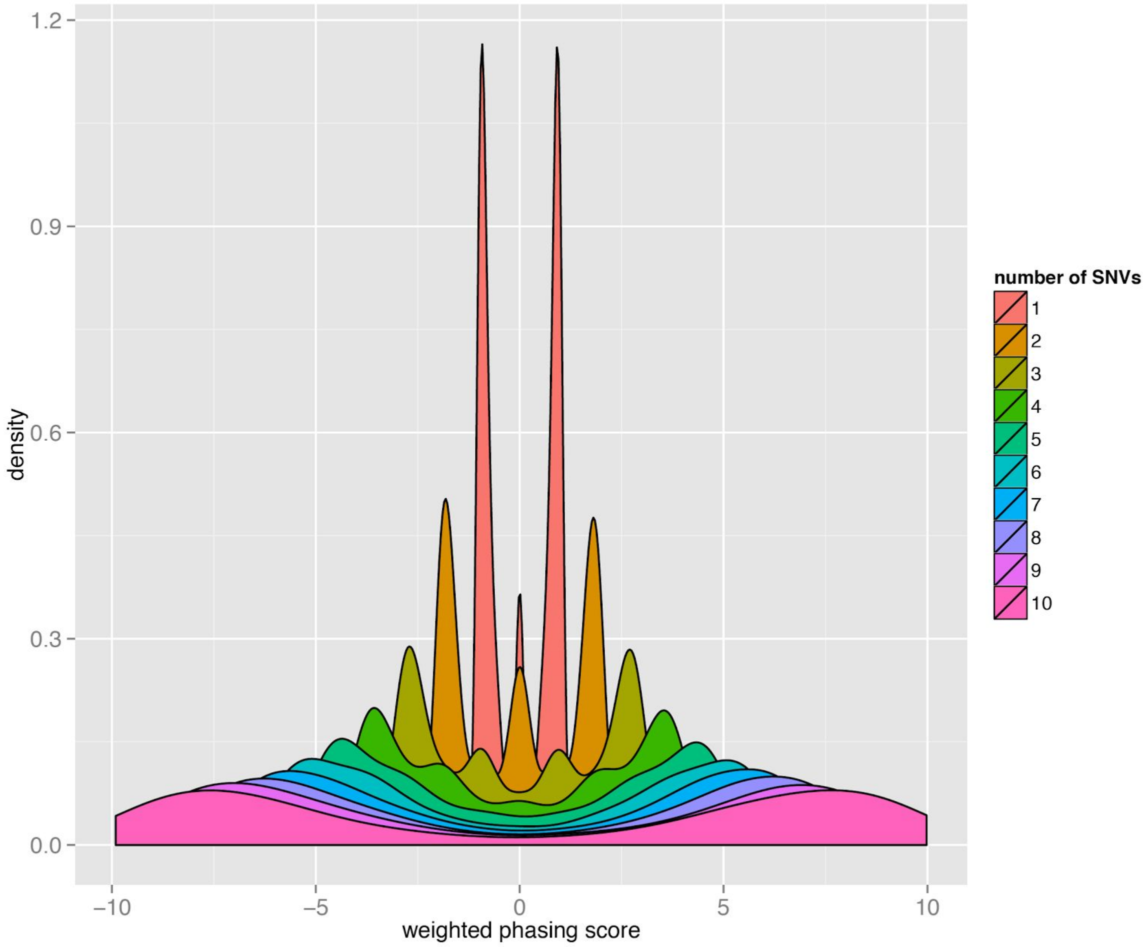

Supplementary Figure 24. Phasing-score distribution for nanopore reads from Patient1.

For each nanopore read a phasing-score *S* was calculated (x-axis, Methods). The plot shows the distribution of phasing scores (*S*) for nanopore reads overlapping 1 to 10 phase-informative SNVs. If the phasing score *S* is positive, the read is assigned to the paternal haplotype, while for a negative value of *S* the read is assigned to the maternal haplotype.

a

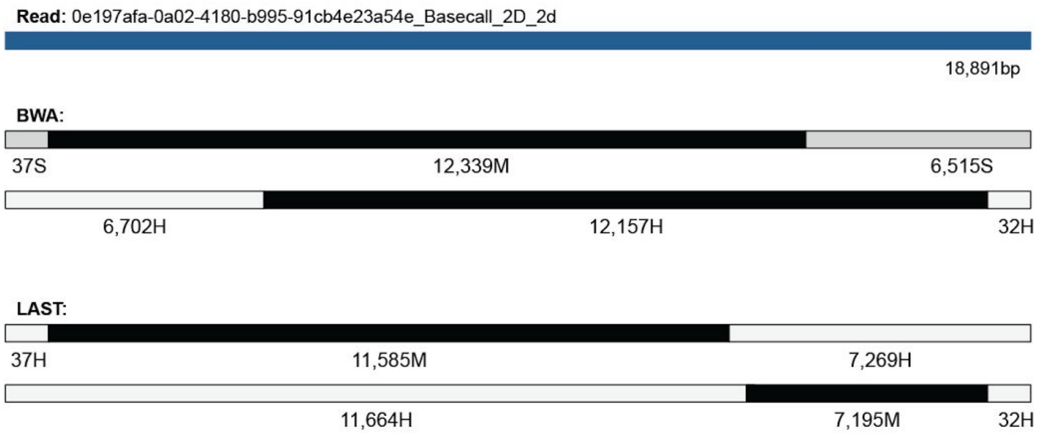

b

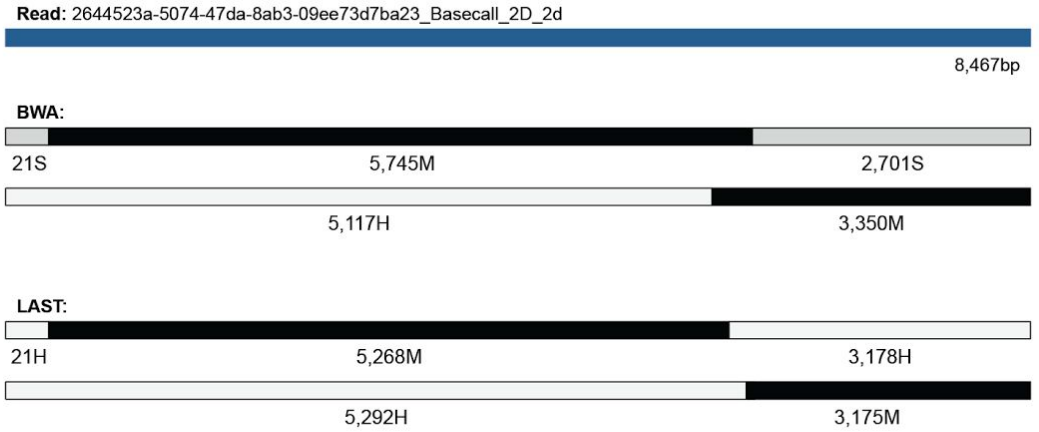

Supplementary Figure 25. Alignment differences between BWA MEM and LAST.
Two examples (**a** and **b**) of how BWA and LAST segment the same read differently at alignment. Each whole read is depicted in blue. For each caller, the two grey/black lines depict how the read is split into two segments at alignment. The black line depicts the part of the read that is aligned and the grey parts depict the clipped parts of the read, for each segment respectively. Both these examples show how bwa splits reads into (at least slightly) overlapping segments, which impair our ability to evaluate candidate breakpoints. Only the read from example **b** contributes to a non HOM_REF SV call in our dataset.

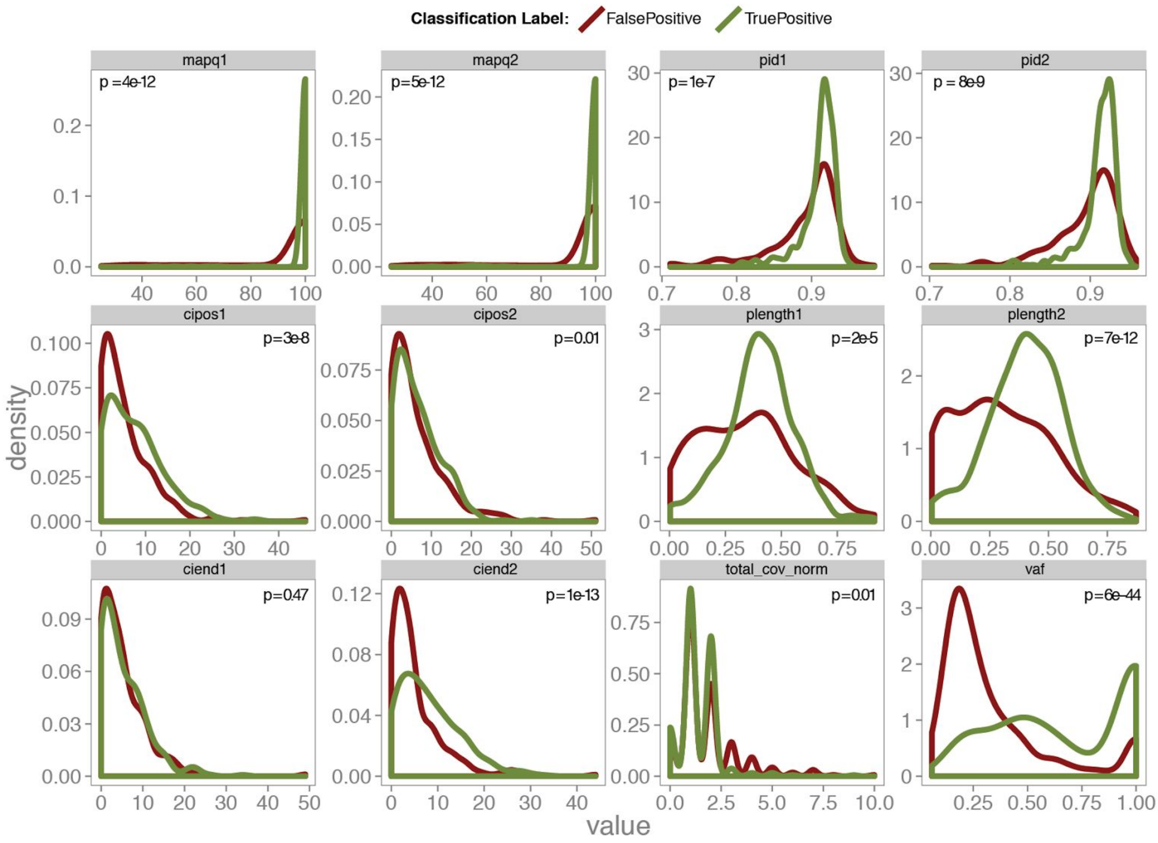

Supplementary Figure 26. Distribution of random forest feature values in the NA12878 data.

Distribution of random forest feature values, within the NA12878 training data, for true positives (green) and false positives (red) respectively. P-values are derived from a two-sided unpaired wilcoxon test.

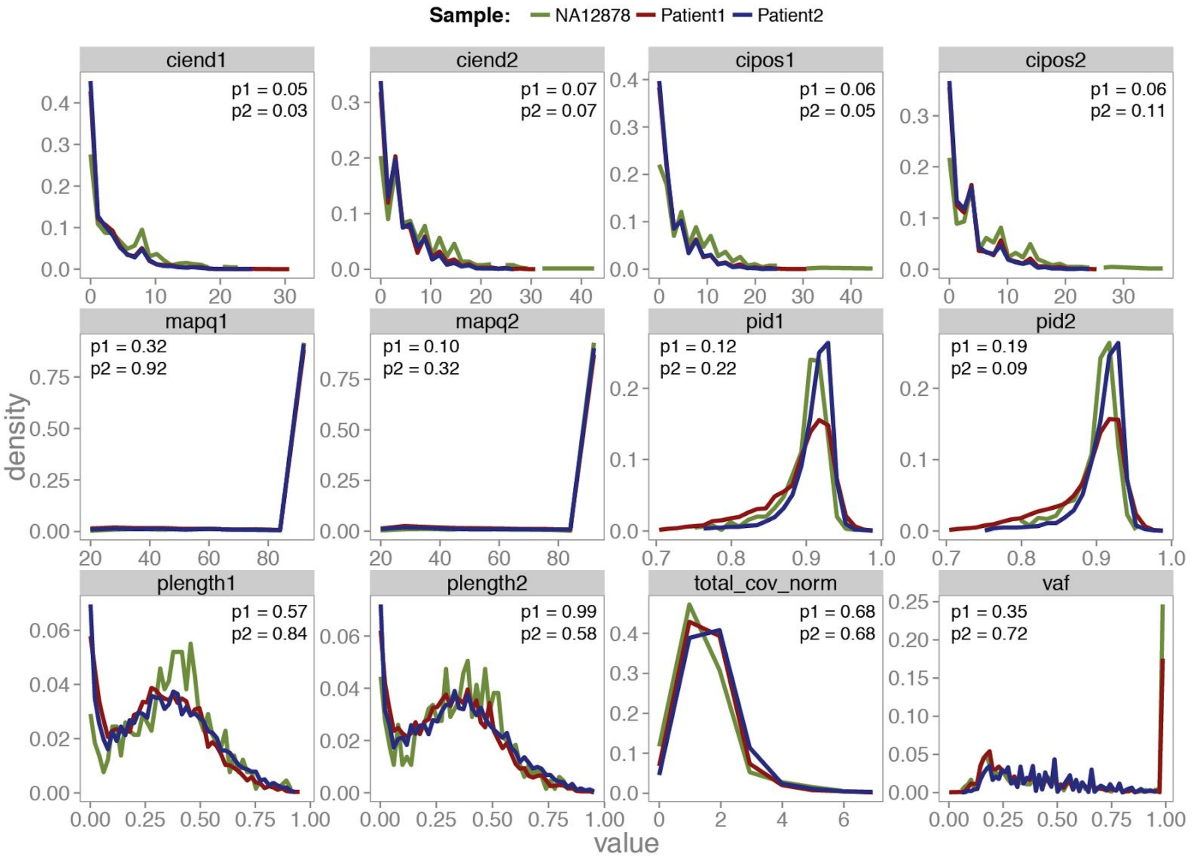

Supplementary Figure 27. Distribution of random forest feature values across samples.

Distribution of random forest feature values across all SV calls (after filtering for homopolymers and simple repeats) within NA12878 (green), Patient1 (red) and Patient2 (blue). The feature distribution of the training data (NA12878) is compared to the feature distribution of the two test samples, Patient1 and Patient2 using a wilcoxon paired test and the two p-values are reported in each feature plot; p-value p1 (comparing NA12878 and Patient1 distributions) and p-value p2 (comparing NA12878 and Patient2 distributions).

Supplementary Table 1. Overview of MinION sequencing runs performed for Patient1 and Patient2.

Supplementary Table 1.							
run_number	run_ID	flowcell_version	flowcell_name	library	library kit	date	MinION
1	Patient1_lib1_1	R7		2D	2D ligation prep	26/04/16	Mk1
2	Patient1_lib1_2	R7		2D	2D ligation prep	26/04/16	Mk1
3	Patient1_lib2_1	R7		2D	2D ligation prep	03/05/16	Mk1
4	Patient1_lib2_2	R7		2D	2D ligation prep	03/05/16	Mk1
5	Patient1_lib2_3	R7		2D	2D ligation prep	03/05/16	Mk1
6	Patient1_lib2_4	R7		2D	2D ligation prep	03/05/16	Mk1
7	Patient1_lib3_1	R7		2D	2D ligation prep	04/05/16	Mk1
8	Patient1_lib3_2	R7		2D	2D ligation prep	04/05/16	Mk1
9	Patient1_lib3_3	R7		2D	2D ligation prep	04/05/16	Mk1
10	Patient1_lib3_4	R7		2D	2D ligation prep	04/05/16	Mk1
11	Patient1_lib4_1	R7		2D	2D ligation prep	09/05/16	Mk1
11	Patient1_lib4_1_restart	R7		2D	2D ligation prep	10/05/16	Mk1
11	Patient1_lib4_1_restart2	R7		2D	2D ligation prep	11/05/16	Mk1
12	Patient1_lib4_2	R7		2D	2D ligation prep	09/05/16	Mk1
12	Patient1_lib4_2_restart	R7		2D	2D ligation prep	10/05/16	Mk1
13	Patient1_lib4_3	R7		2D	2D ligation prep	09/05/16	Mk1
14	Patient1_lib4_4	R7		2D	2D ligation prep	09/05/16	Mk1
15	Patient1_lib5_1	R7		2D	2D ligation prep	11/05/16	Mk1
15	Patient1_lib5_1_restart	R7		2D	2D ligation prep	12/05/16	Mk1
16	Patient1_lib5_2	R7		2D	2D ligation prep	11/05/16	Mk1
17	Patient1_lib5_3	R7		2D	2D ligation prep	11/05/16	Mk1
17	Patient1_lib5_3_restart	R7		2D	2D ligation prep	12/05/16	Mk1
18	Patient1_lib5_4	R7		2D	2D ligation prep	11/05/16	Mk1
19	Patient1_lib6_1	R7		2D	2D ligation prep	23/05/16	Mk1
20	Patient1_lib6_2	R7		2D	2D ligation prep	23/05/16	Mk1
21	Patient1_lib7_1	R7		2D	2D ligation prep	26/05/16	Mk1
22	Patient1_lib7_2	R7		2D	2D ligation prep	26/05/16	Mk1
23	Patient1_lib8_1	R7		2D	2D ligation prep	30/05/16	Mk1B
24	Patient1_lib8_2	R7		2D	2D ligation prep	30/05/16	Mk1
25	Patient1_lib9_1	R7		2D	2D ligation prep	31/05/16	Mk1
26	Patient1_lib9_2	R7		2D	2D ligation prep	31/05/16	Mk1
27	Patient1_lib10_1	R7		2D	2D ligation prep	01/06/16	Mk1B
28	Patient1_lib11_1	R7		2D	2D ligation prep	06/06/16	Mk1
29	Patient1_lib11_2	R7		2D	2D ligation prep	06/06/16	Mk1B
30	Patient1_lib11_3	R7		2D	2D ligation prep	06/06/16	Mk1
31	Patient1_lib11_4	R7		2D	2D ligation prep	06/06/16	Mk1
32	Patient1_lib12_1	R7		2D	2D ligation prep	08/06/16	Mk1
33	Patient1_lib12_2	R7		2D	2D ligation prep	08/06/16	Mk1
34	Patient1_lib12_3	R7		2D	2D ligation prep	08/06/16	Mk1B
35	Patient1_lib12_4	R7		2D	2D ligation prep	08/06/16	Mk1
36	Patient1_lib12_5	R7		2D	2D ligation prep	08/06/16	Mk1
37	Patient1_lib13_1	R7		2D	2D ligation prep	13/06/16	Mk1
38	Patient1_lib13_2	R7		2D	2D ligation prep	13/06/16	Mk1
39	Patient1_lib13_3	R7		2D	2D ligation prep	13/06/16	Mk1B
40	Patient1_lib13_4	R7		2D	2D ligation prep	13/06/16	Mk1
41	Patient1_lib13_5	R7		2D	2D ligation prep	13/06/16	Mk1
42	Patient1_lib14_1	R9		2D	2D ligation prep	16/06/16	Mk1
43	Patient1_lib14_2	R9		2D	2D ligation prep	16/06/16	Mk1
44	Patient1_lib14_3	R9		2D	2D ligation prep	16/06/16	Mk1B
45	Patient1_lib14_4	R9		2D	2D ligation prep	16/06/16	Mk1
46	Patient1_lib14_5	R9		2D	2D ligation prep	16/06/16	Mk1
47	Patient1_lib15_1	R9		2D	2D ligation prep	21/06/16	Mk1
48	Patient1_lib15_2	R9		2D	2D ligation prep	21/06/16	Mk1
49	Patient1_lib15_3	R9		2D	2D ligation prep	21/06/16	Mk1
50	Patient1_lib15_4	R9		2D	2D ligation prep	21/06/16	Mk1B
51	Patient1_lib15_5	R9		2D	2D ligation prep	21/06/16	Mk1
52	Patient1_lib15_6	R9		2D	2D ligation prep	21/06/16	Mk1
53	Patient1_lib16_1	R7		2D	2D ligation prep	23/06/16	Mk1
54	Patient1_lib16_2	R7		2D	2D ligation prep	23/06/16	Mk1B
54	Patient1_lib16_2re	R7		2D	2D ligation prep	23/06/16	Mk1B
55	Patient1_lib16_3	R7		2D	2D ligation prep	23/06/16	Mk1
56	Patient1_lib17_1	R9		2D	2D ligation prep	01/07/16	Mk1B
57	Patient1_lib17_2	R9		2D	2D ligation prep	01/07/16	Mk1B
58	Patient1_lib18_1	R9		2D	2D ligation prep	04/07/16	Mk1B
59	Patient1_lib18_2	R9		2D	2D ligation prep	04/07/16	Mk1B
60	Patient1_lib18_3	R9		2D	2D ligation prep	04/07/16	Mk1B
61	Patient1_lib18_4	R9		2D	2D ligation prep	04/07/16	Mk1B
62	Patient1_lib18_5	R9		2D	2D ligation prep	04/07/16	Mk1B
63	Patient1_lib18_6	R9		2D	2D ligation prep	04/07/16	Mk1B

64	Patient1_lib19_1	R9		2D	2D ligation prep	07/07/16	Mk1B
65	Patient1_lib19_2	R9		2D	2D ligation prep	07/07/16	Mk1B
66	Patient1_lib19_3	R9		2D	2D ligation prep	07/07/16	Mk1B
67	Patient1_lib19_4	R9		2D	2D ligation prep	07/07/16	Mk1B
68	Patient1_lib19_5	R9		2D	2D ligation prep	07/07/16	Mk1B
69	Patient1_lib20_1	R9		2D	2D ligation prep	12/07/16	Mk1B
70	Patient1_lib20_2	R9		2D	2D ligation prep	12/07/16	Mk1B
71	Patient1_lib20_3	R9		2D	2D ligation prep	12/07/16	Mk1B
72	Patient1_lib20_4	R9		2D	2D ligation prep	12/07/16	Mk1B
73	Patient1_lib20_5	R9		2D	2D ligation prep	12/07/16	Mk1B
74	Patient1_lib20_6	R9		2D	2D ligation prep	12/07/16	Mk1B
75	Patient1_lib21_1	R9		2D	2D ligation prep	14/07/16	Mk1B
76	Patient1_lib21_2	R9		2D	2D ligation prep	14/07/16	Mk1B
77	Patient1_lib21_3	R9		2D	2D ligation prep	14/07/16	Mk1B
78	Patient1_lib21_4	R9		2D	2D ligation prep	14/07/16	Mk1B
79	Patient1_lib21_5	R9		2D	2D ligation prep	14/07/16	Mk1B
80	Patient1_lib21_6	R9		2D	2D ligation prep	14/07/16	Mk1B
81	Patient1_lib23_1	R9		2D	2D ligation prep	15/07/16	Mk1B
82	Patient1_lib24_1	R9		2D	2D ligation prep	19/07/16	Mk1B
83	Patient1_lib25_1	R9		2D	2D ligation prep	21/07/16	Mk1B
84	Patient1_lib25_2	R9		2D	2D ligation prep	21/07/16	Mk1B
85	Patient1_lib26_1	R9		2D	2D ligation prep	25/07/16	Mk1B
86	Patient1_lib26_2	R9		2D	2D ligation prep	25/07/16	Mk1B
87	Patient1_lib26_3	R9		2D	2D ligation prep	25/07/16	Mk1B
88	Patient1_lib26_4	R9		2D	2D ligation prep	25/07/16	Mk1B
89	Patient1_lib26_5	R9		2D	2D ligation prep	25/07/16	Mk1B
85	Patient1_lib26_1_refill	R9		2D	2D ligation prep	26/07/16	Mk1B
86	Patient1_lib26_2_refill	R9		2D	2D ligation prep	26/07/16	Mk1B
87	Patient1_lib26_3_refill	R9		2D	2D ligation prep	26/07/16	Mk1B
88	Patient1_lib26_4_refill	R9		2D	2D ligation prep	26/07/16	Mk1B
89	Patient1_lib26_5_refill	R9		2D	2D ligation prep	26/07/16	Mk1B
90	Patient1_lib27_1	R9		2D	2D ligation prep	27/07/16	Mk1B
91	Patient1_lib27_2	R9		2D	2D ligation prep	27/07/16	Mk1B
92	Patient1_lib27_3	R9		2D	2D ligation prep	27/07/16	Mk1B
93	Patient1_lib27_4	R9		2D	2D ligation prep	27/07/16	Mk1B
94	Patient1_lib27_5	R9		2D	2D ligation prep	27/07/16	Mk1B
95	Patient1_sheared_ss	R9		2D	2D ligation prep	31/08/16	Mk1B
96	Patient1_shearedWK	R9		2D	2D ligation prep	31/08/16	Mk1B
97	Patient1_sheared_ss_run2	R9		2D	2D ligation prep	01/09/16	Mk1B
98	Patient1_9_20kb_rapidprep_	R9		1D	1D rapid prep	07/09/16	Mk1B
99	Patient1_9_rapidprep	R9		1D	1D rapid prep	07/09/16	Mk1B
100	Patient1_9_rapidprep_lib2	R9		1D	1D rapid prep	14/09/16	Mk1B
101	Patient1_8_rapidprep_lib2	R9		1D	1D rapid prep	14/09/16	Mk1B
102	Patient1_10_3_1	R9		1D	1D rapid prep	21/09/16	Mk1B
103	Patient1_10_3_2	R9		1D	1D rapid prep	21/09/16	Mk1B
104	Patient1_10_2	R9		1D	1D rapid prep	21/09/16	Mk1B
105	Patient1_10_1	R9		1D	1D rapid prep	21/09/16	Mk1B
106	Patient1_10_4_1	R9		1D	1D rapid prep	22/09/16	Mk1B
107	Patient1_10_4_2	R9		1D	1D rapid prep	22/09/16	Mk1B
108	Patient1_10_4_3	R9		1D	1D rapid prep	22/09/16	Mk1B
109	Patient1_10_5_1_rapid	R9		1D	1D rapid prep	26/09/16	Mk1B
110	Patient1_10_5_2_rapid	R9		1D	1D rapid prep	26/09/16	Mk1B
111	Patient1_lib28_1	R9		2D	2D ligation prep	27/09/16	Mk1B
112	Patient1_lib29_1	R9		2D	2D ligation prep	27/09/16	Mk1B
113	Patient1_lib29_2	R9		2D	2D ligation prep	27/09/16	Mk1B
114	Patient1_lib30_1	R9		2D	2D ligation prep	28/09/16	Mk1B
115	Patient1_lib31_1	R9.4	FLO-MIN106	2D	2D ligation prep	24/10/16	Mk1B
116	Patient1_lib31_2	R9		2D	2D ligation prep	24/10/16	Mk1B
117	Patient1_lib31_3	R9		2D	2D ligation prep	24/10/16	Mk1B
118	Patient1_lib32_1_1D	R9.4	FLO-MIN106	1D ligation	1D ligation prep	14/11/2016	Mk1B
119	Patient1_lib32_1_1D_size_select	R9.4	FLO-MIN106	1D ligation	1D ligation prep	15/11/2016	Mk1B
120	Patient1_lib33_1_1D	R9.4	FLO-MIN106	1D ligation	1D ligation prep	15/11/2016	Mk1B
121	Patient1_X_Rapid_1	R9.4	FLO-MIN106	1D rapid	1D ligation prep	15/11/2016	Mk1B
122	Patient1_lib33_2_1D	R9.4	FLO-MIN106	1D ligation	1D ligation prep	15/11/2016	Mk1B
123	Patient2_lib1_2D	R9.4	FLO-MIN106	2D ligation	2D ligation prep	24/11/16	Mk1B
124	Patient2_lib2_1D	R9.4	FLO-MIN106	1D ligation	1D ligation prep	28/11/16	Mk1B
125	Patient2_lib2_2_1D	R9.4	FLO-MIN106	1D ligation	1D ligation prep	28/11/16	Mk1B
126	Patient2_lib3_1_1D	R9.4	FLO-MIN106	1D ligation	1D ligation prep	29/11/16	Mk1B
127	Patient2_lib3_2_1D	R9.4	FLO-MIN106	1D ligation	1D ligation prep	29/11/16	Mk1B
128	Patient2_lib4_1_1D	R9.4	FLO-MIN106	1D ligation	1D ligation prep	30/11/16	Mk1B
129	Patient2_lib4_2_1D	R9.4	FLO-MIN106	1D ligation	1D ligation prep	30/11/16	Mk1B
130	Patient2_lib5_1_1D	R9.4	FLO-MIN106	1D ligation	1D ligation prep	05/12/16	Mk1B
131	Patient2_lib5_2_1D	R9.4	FLO-MIN106	1D ligation	1D ligation prep	05/12/16	Mk1B
132	Patient2_lib6_1_1D	R9.4	FLO-MIN106	1D ligation	1D ligation prep	06/12/16	Mk1B
133	Patient2_lib6_2_1D	R9.4	FLO-MIN106	1D ligation	1D ligation prep	06/12/16	Mk1B

134	Patient2_lib7_1_1D	R9.4	FLO-MIN106	1D ligation	1D ligation prep	07/12/16	Mk1B
135	Patient2_lib7_2_1D	R9.4	FLO-MIN106	1D ligation	1D ligation prep	07/12/16	Mk1B

Supplementary Table 2. Overview of chromothripsis breakpoint-junctions in Patient1 and Patient2.

									caller overlap						
Patient ID	Sex	Reported Karyotype	Breakpoint Junction ID	Chr1	Pos1	Chr2	Pos2	Orientation	nanosv	lumpy	sniffles	manta	delly	sanger validation	
Patient 1	F	46,XX,ins(2;9)(q24.3;p22.1p24.3)dn	id1	2	181265670	7	149006956	HH	5	5	5	5	5	yes	
			id2	2	176643961	8	57149801	HT	5	5	0	5	5	yes	
			id3	9	13643427	9	32391692	HH	4	4	4	4	4	yes	
			id4	9	24354925	9	33924070	HH	4	0	4	4	4	yes	
			id5	9	25553959	9	30707344	HH	4	4	4	4	4	yes	
			id6	2	167393746	2	175139580	HH	4	4	4	4	4	yes	
			id7	2	167404983	2	174873171	HH	4	4	4	4	4	yes	
			id8	2	167842499	2	175197757	HH	4	4	4	4	4	yes	
			id9	2	176751445	2	176857276	HH	4	4	4	4	4	yes	
			id10	2	180681362	2	180737225	HH	4	4	4	4	4	yes	
			id11	2	167032703	9	32809747	TT	5	0	0	5	5	yes	
			id12	9	16355040	9	30707343	HT	2	2	2	2	2	yes	
			id13	9	30571114	9	38487439	HT	2	2	2	2	2	yes	
			id14	8	58906797	9	30076490	HT	5	5	0	5	5	yes	
			id15	8	59535603	8	59767519	HH	4	4	4	4	4	yes	
			id16	2	181096216	8	57149796	TH	5	5	0	5	5	yes	
			id17	2	174582947	2	175479073	TH	1	1	1	1	1	yes	
			id18	2	180681358	2	181414519	TH	1	0	1	1	1	yes	
			id19	2	174340626	8	58906788	TT	5	5	5	5	5	yes	
			id20	2	174445536	8	58550184	TT	5	0	0	5	5	yes	
			id21	2	176751445	8	59767522	TT	5	0	0	5	5	yes	
			id22	2	167032588	2	175479070	TT	4	4	4	4	4	yes	
			id23	2	167404982	2	181414520	TT	4	4	4	4	4	yes	
			id24	2	167842499	2	175139578	TT	4	0	0	4	4	yes	
			id25	2	174873089	2	181265671	TT	4	4	4	4	4	yes	
			id26	2	176643950	2	176857275	TT	4	4	4	4	4	yes	
			id27	2	181096226	9	32391693	HT	5	5	5	5	5	yes	
			id28	2	175197731	9	14497726	TH	5	5	5	5	5	yes	
			id29	9	14497718	9	33924067	TT	4	4	4	4	4	yes	
			id30	9	25553961	9	30570814	TT	4	4	4	4	4	yes	
			id31	2	167032588	9	30076491	HH	5	0	5	5	5	yes	
			id32	2	174445538	9	15353188	HH	5	5	5	5	5	yes	
			id33	9	13643428	9	32809746	TH	1	1	1	1	1	yes	
			id34	9	16355037	9	37701784	TH	1	1	1	1	1	yes	
			id35	9	37701783	9	38487488	TH	1	1	1	1	1	yes	
			id36	7	149006954	9	15353186	TT	5	5	5	5	5	yes	
			id37	2	174582948	2	174894150	HT	2	2	2	2	2	yes	
			id38	2	174894154	2	180737215	HT	2	2	2	2	2	yes	
			id39	2	174340639	8	58550183	HH	5	5	0	5	5	yes	
			id40	8	59535601	9	24354926	TT	5	5	0	5	5	yes	
Patient ID	Sex	Reported Karyotype	BreakpointJunction ID	Chr1	Pos1	Chr2	Pos2	Orientation	delly	manta	sniffles	lumpy	nanosv	Redin	sanger validation
Patient 2	M	46,XY,t(1;9;5)(complex)dn	id1	9	15012145	9	34091571	TT	4	4	4	4	4	4	yes
			id2	9	13513244	9	14228545	HT	2	2	2	2	2	2	yes
			id3	9	13264315	9	30099291	HT	2	2	0	0	2	2	yes
			id4	9	13242779	9	13522348	TT	4	4	4	4	4	4	yes
			id5	9	13218885	9	13242778	TH	1	1	1	1	1	1	yes
			id6	9	12672158	9	13183542	TT	4	4	0	0	4	4	yes
			id7	9	12648848	9	13525736	HT	2	2	0	0	2	2	yes
			id8	9	12648817	9	14528059	TT	4	4	0	0	4	4	yes
			id9	9	12606162	9	13183544	HH	4	4	4	4	4	4	yes
			id10	9	12606167	9	13264316	TT	4	4	0	0	4	4	yes
			id11	5	105819596	9	22144161	HT	5	5	0	0	5	5	yes
			id12	5	105819379	9	13218889	TH	5	5	0	5	5	5	yes
			id13	1	231543921	9	14469693	TT	5	5	5	0	5	5	yes
			id14	9	15593110	9	22144183	HH	4	4	4	4	4	4	no
			id15	9	13525758	9	30099287	HH	0	0	4	4	4	4	yes
			id16	9	12640136	9	34091605	TH	1	0	1	0	1	1	yes
			id17	9	13853145	9	28554868	HT	2	2	0	0	2	2	yes
			id18	9	14469734	9	15318845	HT	2	0	2	0	2	2	yes
			id19	9	12672157	9	13260301	HT	2	2	2	2	2	2	no
			id20	9	13260300	9	28554865	HH	0	0	4	0	4	4	yes
			id21	9	12640145	9	13853141	HT	0	0	2	0	2	2	yes
			id22	1	231544018	9	14228573	HH	0	0	0	0	5	5	yes
			id23	9	6727607	9	30688945	TH	0	0	0	0	1	1	yes
			id24	9	6728095	9	30690055	HT	0	0	0	0	0	2	no
			id25	9	14528095	9	13613209	HT	2	2	0	0	2	0	yes
			id26	9	13613119	9	14468237	HH	0	0	0	0	4	0	yes

			id27	9	14468307	9	15012828	TH	1	0	0	0	1	0	yes
			id28	9	15318893	9	27997913	HT	0	0	2	0	2	2	no
			id29	9	14467216	9	15012817	HT	2	2	2	0	2	0	yes
			id30	9	15012738	9	15593114	HT	2	2	2	0	2	0	yes
			id31	9	13612748	9	27998195	TH	0	0	0	0	0	1	no
			id32	9	13613355	9	14466984	HT	0	0	0	0	0	2	not tested
**Orientation**	**H=head; T=tail**														
**Caller overlap**	**0=no overlap; 1-5=overlap; numbers indicate type of breakpoint junction as derived from the breakpoint orientation and coordinates**														

Supplementary References

1. Yang, C., Chu, J., Warren, R. L. & Birol, I. NanoSim: nanopore sequence read simulator based on statistical characterization. *Gigascience* (2017). doi:10.1093/gigascience/gix010
2. Layer, R. M., Chiang, C., Quinlan, A. R. & Hall, I. M. LUMPY: a probabilistic framework for structural variant discovery. *Genome Biol.* **15,** R84 (2014).
3. Sedlazeck, F. J. *et al.* Accurate detection of complex structural variations using single molecule sequencing. (2017). doi:10.1101/169557
4. Redin, C. *et al.* The genomic landscape of balanced cytogenetic abnormalities associated with human congenital anomalies. *Nat. Genet.* (2016). doi:10.1038/ng.3720
5. Li, H. Minimap and miniasm: fast mapping and de novo assembly for noisy long sequences. *Bioinformatics* **32,** 2103–2110 (2016).
6. Sudmant, P. H. *et al.* An integrated map of structural variation in 2,504 human genomes. *Nature* **526,** 75–81 (2015).
